# Exploiting the recognition code for elucidating the mechanism of zinc finger protein-DNA interactions

**DOI:** 10.1186/s12864-016-3324-8

**Published:** 2016-12-22

**Authors:** Shayoni Dutta, Spandan Madan, Durai Sundar

**Affiliations:** Department of Biochemical Engineering and Biotechnology, DBT-AIST International Laboratory for Advanced Biomedicine (DAILAB), Indian Institute of Technology Delhi, New Delhi, 110016 India

## Abstract

**Background:**

Engineering zinc finger protein motifs for specific binding to double-stranded DNA is critical for targeted genome editing. Most existing tools for predicting DNA-binding specificity in zinc fingers are trained on data obtained from naturally occurring proteins, thereby skewing the predictions. Moreover, these mostly neglect the cooperativity exhibited by zinc fingers.

**Methods:**

Here, we present an ab-initio method that is based on mutation of the key α-helical residues of individual fingers of the parent template for Zif-268 and its consensus sequence (PDB ID: 1AAY). In an attempt to elucidate the mechanism of zinc finger protein-DNA interactions, we evaluated and compared three approaches, differing in the amino acid mutations introduced in the Zif-268 parent template, and the mode of binding they try to mimic, i.e., modular and synergistic mode of binding.

**Results:**

Comparative evaluation of the three strategies reveals that the synergistic mode of binding appears to mimic the ideal mechanism of DNA-zinc finger protein binding. Analysis of the predictions made by all three strategies indicate strong dependence of zinc finger binding specificity on the amino acid propensity and the position of a 3-bp DNA sub-site in the target DNA sequence. Moreover, the binding affinity of the individual zinc fingers was found to increase in the order Finger 1 < Finger 2 < Finger 3, thus confirming the cooperative effect.

**Conclusions:**

Our analysis offers novel insights into the prediction of ZFPs for target DNA sequences and the approaches have been made available as an easy to use web server at http://web.iitd.ac.in/~sundar/zifpredict_ihbe

**Electronic supplementary material:**

The online version of this article (doi:10.1186/s12864-016-3324-8) contains supplementary material, which is available to authorized users.

## Background

### Zinc finger engineering

The field of targeted genome engineering is still incipient, and, there is a compelling need to develop tools which can meet the ever growing requirements of the field: designing DNA templates of our choice, construction and manipulation of DNA sequences, and tools for the implementation, testing and debugging of genome editing experiments. The rationale to study about zinc finger domain and its interaction with the DNA stems from the need to expatiate on the mechanisms by which the binding of transcription activators and repressors to the genome regulates the expression repertoire of all genes in a cell, hence focussing on its enormous scope in genome engineering.

To exploit zinc finger proteins for genome manipulation, molecular and structural insights at the binding interface of zinc fingers and corresponding DNA targets are mandatory. The most common DNA-binding motif found in humans and multicellular organism is the cysteine-histidine (Cys2-His2) zinc finger. As reported in the literature, the complementing structures of ZFPs and their corresponding DNA binding domains make these systems highly conducive for designing artificial DNA binding proteins [1, 2]. The recognition site for a ZFP motif is primarily composed of a three-nucleotide sequence triplet within the DNA substrate and the recognition specificity is strongly dependent on the amino acids located at positions −1, +1, +2, +3, +4, +5 and +6, relative to the start position of the alpha helix. While the remaining residues form a conserved backbone of the ZFP, any changes in the variable residues, specifically, at the positions −1, 2, 3 and 6 are expected to have a much more pronounced impact on the binding specificity of the ZFP, as compared to any changes in the residues forming the conserved backbone.

Although there is no simple, general code for zinc finger protein–DNA recognition, selection strategies have been developed that allow these proteins to be designed to target almost any desired site on double-stranded DNA. The Cys2His2 zinc finger proteins, and more often, Zif-268, offers a stable and versatile framework for the design of such proteins [1]. This motif has been found to be occurring in a large number of natural proteins that recognize specific DNA sites. Also, the covalent linkage of multiple DNA-binding domains and the fact that these proteins do not require symmetric binding sites offers a practical advantage in the designing of ZFPs derived from mutations in the Zif-268 protein template. The importance of designing ZFPs which bind to target DNA sequences is further aided by the developments in chemical methods for protein synthesis that enables the preparation of zinc finger proteins containing amino acids that do not usually occur in these proteins. To accurately engineer such designer ZFPs, computational tools exploring binding affinity and specificity are extremely significant [3, 4].

The affinity and specificity of these new proteins can also be improved by linking multiple fingers together or by designing proteins that bind as dimers and thus recognize an extended site on the DNA. These new proteins can further be modified by adding other domains for the activation or repression of transcription, for DNA cleavage, or silencing through methylation [5, 6]. Needless to say, such designer transcription factors and other new proteins will have important applications in biomedical research and in gene therapy in the years to come.

### ZFP-DNA binding affinity vs. specificity to aid prediction

Measurements of the affinity and specificity of a synthetically designed zinc finger protein can help in evaluating both the potential utility for biological applications and the efficiency of the design/selection process. Dissociation constants, typically measured by gel shift experiments, are reported in studies involving zinc finger protein–DNA complexes and offer a rough standard for the comparison of results. Another measure of specificity includes the comparison between the binding constant at the desired ZFP target site to that of the binding constant at various “mutant” sites wrt DNA. Such experiments typically show a two to ten-fold increase in the K_d_ value for each single base-pair change in the binding site, with mutations near the centre of the binding site usually having larger effects than mutations near the periphery. These side directed mutational studies may aid computational analysis of the binding affinity altered by mutations of choice in the protein template. However, a ZFP with the highest affinity for a particular DNA site need not bind that site with the highest degree of specificity. These factors have important implications on the design of ZFPs with favourable DNA binding specificities and thus, highlight the importance of incorporating affinity, specificity, and environmental requirements in the design process as a whole [7].

As highlighted above, certain factors influencing the DNA-ZFP binding specificity are unpredictable. With binding not being solely dependent on the affinity, the possibility of the ZFP binding to an unwanted DNA site crops up. One strategy to account for this is to increase the length of the DNA sequence targeted, thus decreasing the probability of an unpredictable binding occurring randomly. A single zinc finger recognizes a 3-bp DNA sub-site, which may occur multiple times in a large genome. On the other hand, three zinc fingers linked together would recognize a DNA sequence of 9-bp in length, which is sufficiently long to constitute a rare address in the human genome [8]. This same approach may even be extrapolated to a conjunction of six zinc fingers, depending upon the stringency of the specificity required.

However, the absence of a standard rubric for predicting putative binding efficiency still plagues most approaches in targeted genome editing [9]. Most prediction methods reported in the literature focus either only on affinity or on specificity, almost completely ignoring the other aspect. Thus, there is a compelling need for an appropriate scoring function to evaluate the DNA-ZFP binding specificity in order to ensure that the predicted protein is the best choice for a given DNA target.

### Hydrogen bonds and energy to estimate interaction energy of ZFP-DNA complex

A simple measure to identify the hydrogen bonds formed in the complex, and to quantify their strengths in terms of interaction energies is desirable for the investigation of ZFP-DNA hydrogen bonded systems. The total interaction energies of hydrogen bonded complexes can be obtained in a super molecular ansatz or through selective structural changes which break only the hydrogen bonds, thus disrupting the evaluation of binding efficiency [10]. In light of the shortcomings in the indicators for binding efficiency mentioned above, the interfacial hydrogen bonding energy was chosen as the scoring function to guide the process of predicting optimal ZFPs for a target DNA sequence. This hydrogen bond energy can be captured mathematically as the equation for the AMBER99 force field with its hydrogen bond energy component, as described in the methods section ahead [11]. Though the AMBER energy function isn’t widely used in the literature, it renders our calculations to be far more accurate. The ability to accurately pick every single mutational change affecting the bonding system makes it much more reliable. Though at the interaction interface other than direct interaction like hydrogen bonds forming the recognition code even indirect interactions contribute to the binding affinity and specificity [12].

### Modes of binding

The success of any computational pipeline predicting binding specificities depends strongly on being able to account for the physico-chemical processes occurring at the molecular level. Of the several hypotheses reported in literature for the mechanism governing DNA-ZFP interactions, we have studied in detail two modes of binding – *Modular* and *Synergistic* modes of binding.

*Modular* mode of binding assumes that the binding affinity for each finger of the protein is independent of the other two fingers. The net energy for the interactions between the target DNA and their respective zinc finger protein is thus, simply calculated by adding the respective energies of each finger and its corresponding 3bp DNA target, for all ZFP-DNA pairs. The advantage of this mode is that each finger can be investigated individually for its positional dependence and amino acid propensity without the effect of the adjoining fingers. However, *modular* binding fails to address the cooperativity in the ZFP-DNA binding i.e. the *synergistic* effect of the binding of a finger on the binding of the subsequent ones.

The *modular* assembly (MA) method of generating engineered zinc finger proteins (ZFPs) was the first practical method for creating custom DNA-binding proteins and has enabled the creation of a myriad of sequence-specific methods and reagents, ushering in a new era of zinc finger-based applications. The approach was first used to develop zinc finger nucleases to cleave endogenous sites. Subsequently, MA has been used widely for many applications. There are plethora of tools reported in the literature that are based on assuming *modular* mode of binding at the molecular level, including - OPEN [13], ZiFiT [14], Zif-Predict [15], ZifBASE [16].

*Modular* assembly assumes that the residues at the key positions −1, 3 and 6 on the finger interact with 3 contiguous base pairs on the target DNA strand. The idea of such a recognition code engendered an interest in the possibility of developing custom made zinc fingers for all the 64 possible 3-bp DNA sub-sites. Once the mechanism underlying the recognition code is better understood, it can be utilized by the combination of rational design and combinatorial methods such as phage display to assemble custom designed multiple zinc fingers mutated at their cardinal residues so as to recognize DNA target of our choice.

In the *synergistic* mode of binding, the synergy between the binding affinities of individual fingers, when present in conjunction, are taken into account. Cross-strand interactions, as well as the concept of cooperativity are taken into consideration. The *synergistic* approach to ascertain the functioning of zinc fingers while interacting with the respective target DNA via their recognition code is expected to be more reliable in mimicking the physico-chemical interactions at the molecular level. Though, in this case, the binding affinities and energies cannot be evaluated for individual fingers.

Computational tools reported in literature which assume the *synergistic* mode of binding are a rarity, though, experimental validation of the same for optimal zinc finger proteins binding to all 16 GNNGNNGNN DNA targets have been reported [17]. The intermolecular contact between positions −1 and 2 in a zinc finger shows heightened levels of synergy, which is called the overlapping 4-bp sub-site and can be accounted for by uncovering the complete recognition code. This sheds some light on the possible networks of contact between the protein and the DNA at the interface in the region of the 4bp overlapping sub-sites. This aspect has been ignored in our study to avoid complication and deem the experiment feasible.

### Physico-chemical v/s computational approaches for the prediction of ZFP-DNA interactions

Although it is stipulated that physico-chemical approaches for ZFP binding site predictions can improve significantly as the number of known ZFP-DNA complexes increase, such algorithms have limited scope currently, with little experimental data available for such approaches. Due to the inherent limitations of homology or sequence based prediction software, more realistic and unbiased, structural approaches to predict ZFP motifs are gaining traction. Keeping these limitations in mind, here we present three different approaches attempting to predict optimal zinc finger proteins for a target DNA sequence. These three differ in terms of - Firstly, the mode of binding assumed between the ZFP and the DNA, and secondly, the nature of the mutations introduced in the template Zif-268 protein.

The scoring function used to measure the binding affinity of a particular ZFP-DNA complex is an energy function known as the Interfacial hydrogen bond energy. The function is based on the hydrogen bond interaction energies of the side chains of amino acids and nucleotides in the 3 (−1, 3 & 6) or 4 (−1, 2, 3 & 6) length recognition helix and the nucleotide triplet respectively, at the protein-DNA interface. It can mathematically be expressed in terms an equation for the AMBER99 force field with its hydrogen bond energy component [18]. The interfacial hydrogen bond energy (IHBE) is calculated for all possible complexes formed by all 64 possible 3-bp DNA sequences and zinc finger proteins generated by introducing mutations in the Zif-268 template protein. For each DNA triplet, the energy value for the recognition helix of the complex formed determines the rank of the corresponding Zif-268 mutant. Thus, the recognition helix preferences for each nucleotide triplet are finger-specific, but cross-strand interactions are not accounted for.

The advantage of such a prediction tool is that it is free of a sampling bias that might be introduced by the limited data available in literature, thus preventing the predictions from being skewed. However, these predictions are subject to the binding model assumed, and other assumptions made during the optimization of the docking experiments. Also, the ansatz used for the calculations is a generic function for hydrogen bonding in various systems, and has not been applied to protein-DNA systems in literature or optimized for modelling these interactions. Moreover, it does not represent the exact interaction energy of the two macromolecules, because it takes into account neither the direct nor the water-mediated hydrogen bonding among other residue side-chains and the main chain, or any other types of interactions. Needless to say, with a better understanding of the binding mechanism and more computational resources available at hand, our approach is expected to improve significantly in the times to come.

## Methods

The scoring function guiding our approach theoretically computes the interfacial hydrogen bond energy (IHBE) for every protein-DNA complex based on the Zif-268 motifs generated by introducing all possible mutations to the template protein molecule. Taking all possibilities into account, the total number of complexes is equal to (64* number of Zif-268 mutants * 3). For each possible DNA triplet, the value of this energy for the recognition helix of each complex determines its rank.

The structure with a particular triplet-recognition helix combination at all three fingers was mutated, such that finger-specific scores could be calculated using just one structure. Because the main chains of the protein and DNA molecules remain fixed, there is no influence of neighbouring fingers on the score. The residues at positions −1, +2, +3 and +6 relative to the alpha-helix in each zinc finger motif are chosen as the recognition helix and are represented by an amino-acid sequence of length four [19]. This basic principle can be tweaked based on the framework and the mode of binding assumed.

We have assumed only the hydrogen bonds involving the atoms N, O and S as donors or acceptors in the side chains of the respective amino acids and bases contribute to DNA binding specificity of the recognition helix (note that the hydrogen bonds must be between an atom from an amino acid and an atom from a nucleotide to contribute to the bond energy). Also, the recognition helix preferences for each nucleotide triplet are finger-specific. Finally, cross-strand interactions are assumed to have a negligible influence [20].

### Mutations made to the Zif-268 template

The versatility and the ubiquity of Zif-268 like zinc finger proteins make it a good candidate for a template molecule to which mutations can be made to introduce DNA-binding specificities in a stable framework. Based upon the amino acid propensity, as seen in the zinc finger proteins reported in the literature, we identified a pool of amino acids that have a higher probability of existing at the key residues (positions −1, 2, 3 and 6) within the recognition helix of the zinc finger protein. As the number of combinatorial possibilities increases exponentially when considering all possible mutants of the Zif-268 protein molecule against all 64 possible 3bp DNA sequences, a smaller pool of amino acids to introduce mutations brings down the order of the problem significantly.

**Fig. 1:**
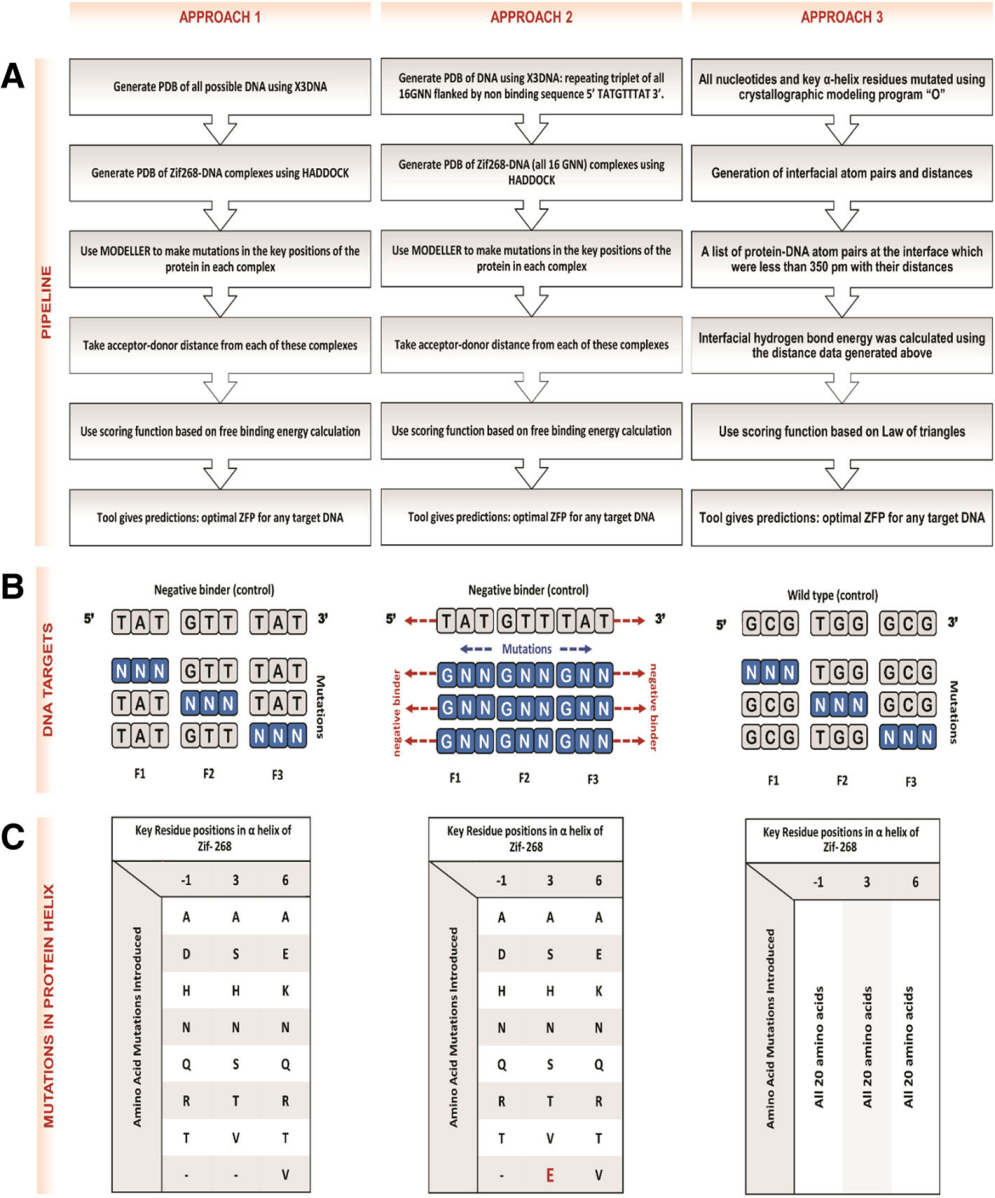
Approaches for predicting binding specificities in zinc finger protein. **a** Pipelines for the three approaches. Prediction of the optimal ZFP for a unique address in the genome is made by searching in the space of Zif-268 mutants in all these approaches. **b** DNA Targets: Approach 1 assumes *modular* mode of binding and a negative binder is used as the control for generating all 64 DNA targets (*N* = A/T/G/C); Approach 2 assumes *synergistic* mode of binding and a negative binder is used both as the control and the flanking unit across the repeating triplets of all possible 16 GNN DNA targets; Approach 3 assumes *modular* mode of binding and the wild type DNA is used as a control for generating all 64 DNA targets. **c** Mutations introduced in the key residues positions of the Zif-268 α-helix: Approach 1 incorporates the consensus amino acid framework as reported by *Isalan* et al. *1998.* [20]; Apart from the mutations introduced in Approach 1, for Approach 2, glutamic acid (E) was introduced at position 3 as well; Approach 3 introduces all 20 amino acids as possibilities at all three positions

Thus, the first method to introduce mutations in the Zif-268 template relies only on amino acids present within this pool (Fig. 1a and b). The advantage of this method is two-fold – firstly, it helps reduce the total number of possibilities that need to be studied by considering only the biologically relevant complexes, which are in sync with the experimental data reported in literature. Secondly, as these complexes are derived from experimentally identified cases, there is ample scope for validation of the ZFP-DNA binding affinities predicted. However, this comes at the cost of forfeiting any chances of identifying Zif-268 mutants from outside the pool of amino acids, which may have had equal or even better specificities than those reported in literature so far.

The second method for introducing mutations goes beyond the pool of amino acids described above - all possible mutations were introduced (Fig. 1c). The advantage in this framework is, as described above, the possibility of uncovering new, unprecedented patterns in zinc finger protein interactions. Moreover, the impact of cross-strand interactions is much more applicable in this case. Cross-strand interactions refer to the interactions between the residues at position 2 of the α-helix with the base on the complementary strand of the target DNA. The two glaring disadvantages of this method are the large number of combinatorial possibilities to be studied, and the high number of false positive predictions.

### Approach 1: modular binding of Zif-268 mutants, derived from a small (consensus) pool of amino acids, to DNA targets

#### Generation of protein and DNA 3-D structures

DNA sequences of the form “NNN-GTT-TAT” for finger 1, “TAT-NNN-TAT” for finger 2 and “TAT-GTT-NNN” for finger 3 were generated, where *N* = A/T/G/C. The 3-D structures of all 64 possible NNN-type (*N* = A/T/G/C) DNA targets for the three fingers were prepared using the 3DNA standalone software package [21]. Using the ‘fiber’ option offered by the 3DNA software package, B-DNA structural models of each sequence were generated, returning the output as a PDB file. Thus, a total of 64*3 = 192 DNA PDBs were generated. Since cross-strand interactions are not being considered in our approach, only a single chain could be used for docking. The protein template used for docking experiments was that of Zif-268 (PDB ID: 1AAY) that was obtained from the Protein Data Bank.

#### Docking of protein

In order to predict the spatial orientation of the two molecules in the protein-DNA complex, each DNA sequence was docked with Zif-268. The algorithm HADDOCK that uses a data-driven approach, was utilized to extract the distance constraints from experimental data (gathered from various possible sources, such as NMR, conservation data, etc.) for reconstruction and refinement of the protein-DNA complex [22].

Docking is the most demanding step in our approach, both in terms of time and the computational resources, and hence, required to be optimized. Thus, here we assumed that the template (Zif-268) and the mutated protein differ at only certain key residues (at most 3 amino acids at the positions −1, +3 and +6 for a particular finger) and hence are not structurally too different. Therefore, in order to get a template complex structure with each DNA sequence, the DNA sequences were docked directly with Zif-268. The numbers of structures for rigid body docking (it0) were from 1000 to 750 and the number of structures for the refinement step (it1) were from 200 to 100 (rate determining step) with the option to randomize starting orientations set to false. The option for solvated rigid body docking was set to false as well.

As the structure for Zif-268 was extracted from an already complexed state with its consensus DNA sequence, it was considered safe to assume that it was close to the conformation it would attain when docked with the new DNA; thereby making it possible for the complex to attain a near optimum spatial arrangement in a lesser number of iterations. The analysis that we conducted is without any solvent. The possible effect of the presence of a solvent like water, which might interfere with the intermolecular hydrogen bonding between DNA and protein, was discarded as it has been shown in literature that the effect of polar solvents on hydrogen bonding in DNA-protein complexes is minimal [23]. Of the numerous structures generated for each DNA-protein (Zif-268) pair, the structure with the greatest HADDOCK score was deemed the most suitable for that pair and further used for the next step in our approach.

#### Mutation of key residues

As discussed above, in this approach, we had introduced mutations using only the amino acids belonging to a small pool that was identified based on amino acid propensity and the positional preference of amino acids in zinc finger proteins reported in literature. Excluding the residues that do not frequently function in DNA recognition helps reduce the library size and the “noise” associated with non-specific binding members of the library. Therefore, the randomizations need not encode all 20 amino acids but rather represent only those residues that are most frequently found to occur in sequence-specific DNA binding at the respective α-helical positions. A list was prepared based on the positional preference of amino acids in zinc fingers, highlighting mutations at key positions that might have a significant effect on the specificity of the interaction [24]. For each DNA sequence target, all possible recognition helices generated by mutating residues at the positions −1, +3, +6 (keeping +2 fixed to eliminate cross strand interactions), a total of 7*7*8 possible recognition helices were complexed with the DNA sequence and finally ranked to identify the helices which best bind the target DNA.

MODELLER is a software package often used for homology or comparative modelling of protein three-dimensional structures. MODELLER was used to introduce the mutations mentioned in Fig. 1c, in the docked complexes formed after the docking step. Then, homology modelling was used to predict the changes made in the structure of the docked complexes, upon introduction of mutations in the Zif-268 protein sequence [25].

#### Scoring metric for binding specificity

One good metric to measure binding affinity, and hence specificity, is to measure the bond energies. The existing energy functions for modelling protein-ligand (and/or protein-protein) interactions may be classified as physico-chemical force fields, empirical scoring functions or knowledge-based statistical potentials. However, computation of binding affinities and interaction for each possible complex using these methods is highly time and resource consuming, making them infeasible for the large number of complexes studied here.

As the purpose here was only to aid the comparison of various Zif-268 mutants as potential binding targets for a particular 9bp DNA target, the exact values of binding affinities were not required. An indirect measure, interfacial hydrogen bond energy, was thus used. It has been shown in literature that the amino acid–base hydrogen bonds are the most prevalent interaction in protein-DNA complexes, accounting for over half the number of bonds [26]. This is followed by van der Waals, hydrophobic and finally, electrostatic interactions. Thus, neglecting other indirect interactions, the desolvation and the deformation kinetics, interfacial hydrogen bond energy can be used as an indicator for the actual overall bond energy for the complexes formed.

A rendition of the equation for the AMBER99 force field with its hydrogen bond energy component was used for measuring hydrogen bond energy, as described ahead. Once the target pairs were identified, the atom types (primarily N or O) of the donor and the acceptor atoms were identified. Using the values of the constants ε_ij_ and d_ij_^’^, as mentioned ahead, the energies were calculated for all possible Zif-268 mutant-DNA target complex. The energy values for all possible helices for a particular DNA codon (and finger) have been reported. Finally, predictions about the optimal zinc finger proteins for a DNA target sequences are made based on these calculated energy values (Fig. 1a).

#### Calculation of the interfacial hydrogen bond energy

Due to the approximate nature of angle-dependent hydrogen bond energy functions reported in the literature, they may not be sensitive enough to pick up on the small differences introduced in the binding affinities due to single point mutation in the recognition helix. One way to improve upon this is to use interfacial hydrogen bond energy as a scoring metric. For calculations, hydrogen bonding parameters like acceptor-donor distance and angles were extracted from the PDBs obtained from MODELLER. For this purpose, the LIGPLOT/HBPLUS software suite was used [27]. For estimation of the interfacial hydrogen bond energy, Eq. 1 was used, as given below (6):

1 $$ \varDelta \mathrm{G}\left(\mathrm{h}\mathrm{b}\right) = {\in}_{ij}\left[3{\left(\frac{{\mathrm{d}}_{ij}^{\hbox{'}}}{{\mathrm{d}}_{ij}}\right)}^8 - 4{\left(\frac{{\mathrm{d}}_{ij}^{\hbox{'}}}{{\mathrm{d}}_{ij}}\right)}^6\right]{ \cos}^4\uptheta $$

where ε_ij_ is the optimum hydrogen-bond energy for the particular hydrogen-bonded atoms i and j, considering that d_ij_^’^is the optimum hydrogen-bond length. ε_ij_ and d_ij_^’^ vary according to the chemical type of the hydrogen-bonded atoms i and j. The values mentioned below were used to quantify the DNA-protein interaction at the interface.

ε_ij_ = 2.0 kcal · mol-1 and d_ij_^’^ = 3.2 Å for N-N hydrogen bonds

ε_ij_ = 2.8 kcal · mol-1 and d_ij_^’^ = 3.0 Å for N-O hydrogen bonds

ε_ij_ = 4.0 kcal · mol-1 and d_ij_^’^ = 2.8 Å for O-O hydrogen bonds [28].

### Approach 2: *synergistic* binding of Zif-268 mutants derived from a small (consensus) pool of amino acids, to DNA targets

As described in the earlier section, *synergistic* binding enables us to incorporate the effect of cooperation in the binding specificities of zinc fingers. In order to better understand the effect of this cooperation, the *modular* approach algorithm described above was modified to incorporate simultaneous binding of three zinc fingers. Thus, the DNA sequence used for docking was 27-bp, instead of 9-bp. The target sequence consists of three 3-bp DNA sub-sites - a binding region flanked by two non-bonding regions. As most zinc finger DNA targets are GC-rich as reported in literature, the binding region was kept GC-rich with flanking non-binding regions (5'-TATGTTTAT -3') (Fig. 1b and c).

Other than the changes in the DNA sequences being studied, approach 2 is exactly the same as approach 1. The mutations made in the Zif-268 template protein are the same in both - drawn from a small pool of amino acids exhibiting high binding affinity for DNA targets, as reported in the literature. The general pipeline is the same for the two approaches, the only difference being in the mode of binding assumed, and thus, the exponential increase in the number of possible complexes in approach 2, as opposed to the previous approach (Fig. 1a).

PDB files for all possible 27-bp long DNA double strands were generated using the ‘fiber’ option of the X3DNA software suite. The generated PDBs for all possible targets in the 27-bp long DNA double strands were docked with the Zif-268 PDB (PDB ID: 1AAY) using HADDOCK. As the total number of possible mutations for each finger was 7*8*8 = 448, hence, the total number of possible complexes formed assuming *synergistic* mode of binding would be 4483, which is computationally infeasible. Thus, we chose to mutate all three fingers simultaneously, thus reducing the total number of mutations. These mutations were introduced using the MODELLER software suite, as in approach 1. The energy was calculated for all possible ZFP-DNA complexes and the most optimal zinc finger proteins were reported for each 27-bp DNA target (Fig. 1a).

### Approach 3: *modular* binding of all possible Zif-268 mutants to DNA targets

#### Mutation of a PDB structure

Unlike approach 1 and 2, in this approach, all possible mutations were introduced in Zif-268. Thus, instead of drawing mutations from a small pool, all 20 amino acids were used. Also, as this approach assumes *modular* mode of binding, each individual zinc finger is assumed to be binding independently to a 3-bp DNA sub-site. This corresponds to a 9bp DNA target sequence consisting of all permutations of the 64 possible 3-bp DNA sub-sites.

After the introduction of the mutations in the cardinal residues of the recognition helix of the bound peptide, and in the corresponding DNA sub-sites for all possible protein-DNA complexes, remodelling of these structures were done using the crystallographic modelling program “O” in order to optimize the relative spatial geometry of the molecules to incorporate the effect of the mutations. For each complex, ‘O’ was used to identify the optimal rotamer for each introduced mutation. Rotamers are preferred orientations for amino acids based upon empirical and stereo-chemical factors of their side chains. A library of rotamers reported in literature was used to determine the structure with the most likely side-chain conformation, thus reducing the time for analysis and generating a more accurate structure. “O” wasn’t used in the previous approaches due to lower number of complexes as compared to this approach; hence more computationally intensive tools were used to assess the complexes in the previous two approaches.

For this purpose, the “penultimate” rotamer library was downloaded and synced with ‘O’ using OOPS to pick the optimal rotamers [29]. These amino acid mutations were introduced using the “mutate_replace” method along with the “on_mutate” macro offered by ‘O’. The process optimization options chosen for this purpose were “lego-loop” and “Lego_Auto_SC” [30]. Finally, the ‘O’ macro was used to write out the generated PDBs for each mutation.

In order to simplify our model, two assumptions were made in modelling side chain conformations: firstly, the backbone conformation remains unchanged upon amino acid replacement owing to which the effects in the backbone can be ignored. Secondly, most side chains in high resolution crystallographic structures can be represented by only a limited number of conformations found in the build-library of rotamers.

### Generation of interfacial atom pairs and distances

A JAVA implementation was used to read the coordinates of all atoms in the recognition helices and triplets from a Zif-268-based PDB structure and generate a list of protein-DNA atom pairs at the interface that were less than 350 pm apart, along with their distances. Atom pairs containing a carbon were ignored. Another JAVA implementation was used to execute the above programs iteratively and produce the final output data.

#### Calculation of interfacial hydrogen bond energy

A JAVA implementation was used to calculate the hydrogen bond energy at the interface using the distance data described above. It determined the probable donor-acceptor pairs from the list of atom-pairs and used their distances for the calculations. If there was more than one atom-pair present for a finger-triplet-recognition helix combination, the individual bond energy contributions were added up to report a sum. The absolute values of the interfacial hydrogen bond energy (kcal/mol) were taken to be the scores that were used to arrange the recognition helix preferences for each nucleotide triplet, finger-wise. For the purpose of validating this approach, a database of nucleotide triplet binding sites for various recognition helices of Zif-268 were retrieved from the literature [31–34]. The wild type Zif-268 binding sites and recognition helices were also included in this database (Fig. 1a and b).

## Results

### Analysis of predictions generated from the three approaches and validation with experimental data

Due to the inherent differences in the mechanisms the three approaches try to mimic, their prediction can differ significantly (Table 1). Here, we have tried to discuss the biological relevance of each approach, and highlight their strengths and their weaknesses. The sensitivity and general accuracy of the interfacial hydrogen bond energy function used was found to be high. Resolution up to the first decimal place was seen even on the introduction of a single point mutation in the amino acid sequences. Also, the selection of the negative control flanking sequences was validated by comparing the energy values of the template 1AAY (ZIf-268 complex with its consensus DNA) and the control complex (Zif-268 complex with negative control DNA) as calculated using HADDOCK. The template complex had energy of −165.5647 units and the control complex had energy of 4.681917 units. The 35-fold difference in their energies clearly validates that the flanking sequences are not interfering with the binding process.

**Table 1:** A matrix to understand the three approaches in terms of the mode of binding used and the pool of amino acids for introducing mutations in the zinc finger template

Mode of Binding	Amino acid mutation	
	Consensus pool of amino acids for introducing mutations in the ZFP	All amino acid possibilities for introducing mutations in the ZFP
*Modular*	Approach 1	Approach 3
*Synergistic*	Approach 2	N.D^a^

^a^ND: not determined due to enormity of the computational complexity

In order to validate our approaches with experimental data reported in the literature, K_d_ values have been compared to our predicted interfacial hydrogen bond energy. DNA sequences reported to be binding with two different zinc finger skeletons (Zif-268 and QNK) have been used to validate the predictions made by approaches assuming *modular* mode of binding, i.e. Approach 1 and Approach 3. Our results show that Approach 1 (consensus amino acids and *modular* binding mode) is found to be performing better for predicting optimal zinc fingers for DNA sequences which experimentally show greater affinity for QNK. While, approach 3 (all possible amino acids and *modular* binding mode) performs well for DNA sequences which have been reported in literature to be binding Zif-268.

As there is little experimental work assuming *synergistic* mode of binding between the zinc finger protein and DNA sequences, validation of approach 2 (consensus amino acids and *synergistic* binding mode) has only been done by comparing the predictions against DNA sequences reported to be binding to the Zif-268 skeleton.

### Approach 1: consensus amino acids and modular binding mode

Validation for this approach was done by comparing the second finger in the zinc finger protein-DNA target pairs identified experimentally, to the predictions made by our approach for the same DNA target sequences. Validation of the predictions shows strong coherence with experimentally reported data. For each of the respective target DNA sequences, the energy values predicted by our approach follow the same trend as the K_d_ values reported in the literature (Table 2). DNA targets reported to be binding to two different zinc finger protein skeletons (Zif-268 and QNK) were used for the validation [35, 36].

**Table 2:** Validation of predictions made by assuming modular mode of binding and mutations from a small consensus pool of amino acids (Approach 1)

Validation with DNA targets binding to QNK			Validation with DNA targets binding to Zif-268		
Target DNA 5' - 3'	Predicted H-bond Energy Finger 2 (QDK)	K_d_ value Jameison et al. 1996 [32]	Target DNA	Predicted H-bond Energy Finger 2 (RHR)	ΔG value Rebar et al. 1994 [33]
GGG **GCG** GAA	2.867	16	GCG **TGG** GCG	0	0
GGG **GCA** GAA	1.064	24	GCG **TAG** GCG	2.18	0.5
GGG **TCA** GAA	0.8568	142	GCG **GGG** GCG	2.15	1.3
GGG **TCG** GAA	0.8477	990	GCG **AGG** GCG	2.77	1.7
GGG **GTC** GAA	0.228	7500	GCG **TTG** GCG	3.22	1.9
GGG **ATC** GAA	0.033	25000	GCG **GAG** GCG	1.19	1.9

The trend of the predicted H-bond energies is similar to the reported Kd values for QNK that bind well to various DNA targets. The trend of the predicted H-bond energies is similar to the reported ΔG values for Zif-268 that binds well to various DNA targets

For this approach, the best predictions were made for the set of DNA sequences which have been reported to be binding well with the QNK skeleton. The energy predictions, and thus the order of affinity was coherent with experimental data, and followed the same trends as the experimentally reported K_d_ values [35]. Also, for the set of DNA sequences reported to be binding well to zinc fingers with the Zif-268 skeleton, barring a few outliers like GGG and GAG, predictions made by the approach are comparable to the experimentally reported K_d_ values, and are thus, reliable.

### Approach 2: consensus amino acids and *synergistic* binding mode

Given the exponential increase in the number of possible complexes formed by a 9-bp DNA sequence and Zif-268 mutants, and since our primary purpose here was to compare how well the predictions made by *synergistic* and *modular* approach match up against experimental data, we restricted the analysis for *synergistic* mode of binding to only 16 GNN triplets, instead of all 64 possible NNN triplets. The predictions were considered to be matching with the zinc finger proteins identified experimentally if the amino acids mutations introduced in the helices of our predictions were similar in terms of polarity and charge. The standard amino acid classification system was used for this purpose. Validation with experimental data proves that approach 2 (consensus amino acids and *synergistic* binding mode) shows great promise and outperforms approach 1 (consensus amino acids and *modular* binding mode). The matches between experimentally identified zinc fingers and the predictions made by approach 2 (consensus amino acids and *synergistic* binding mode) are reported and highlighted in Table 3 [17].

**Table 3:** Validation of predictions by assuming *modular* mode of binding and mutations using all 20 amino acids (Approach 3). Helices predicted for 9-bp repeating triplet of all 16GNN DNA targets are reported. The predictions are exact matches to the experimentally determined helices for the 16 GNN targets earlier reported by Qiang et al. 2002 [17]

Target DNA 5' - 3' Qiang et al. 2002 [17]	F1	F2	F3
	−11234567	−11234567	−11234567
GGG GGG GGG	NSGELTE	RSGTLTR	**RSGSLTR**
GTA GTA GTA	RSGDLTA	NSGDLTR	**QSGTLTT**
GGA GGA GGA	RSGELTK	**NSGNLTR**	**HSGTLTN**
GCT GCT GCT	HSGVLTR	NSGTLTQ	HSGSLTA
GGC GGC GGC	RSGALTR	RSGVLTT	**TSGHLTQ**
GAG GAG GAG	*NSGTLTA*	*TSGTLTR*	*RSGTLTQ*
GCG GCG GCG	HSGDLTR	**RSGSLTR**	QSGSLTT
GAC GAC GAC	HSGHLTR	**QSGTLTT**	QSGVLTR
GGT GGT GGT	DSGNLTQ	RSGALTA	**RSGSLTR**
GTC GTC GTC	HSGNLTQ	**HSGSLTR**	HSGELTQ
GCC GCC GCC	TSGVLTT	RSGTLTA	RSGVLTK
GCA GCA GCA	DSGSLTR	**RSGNLTK**	ASGVLTK
GTG GTG GTG	NSGELTT	RSGHLTT	TSGSLTR
GAT GAT GAT	**HSGALTT**	NSGDLTR	-
GTT GTT GTT	NSGSLTK	**ASGHLTT**	TSGELTQ
GAA GAA GAA	DSGNLTT	DSGNLTT	DSGNLTT

### Approach 3: all possible amino acids and *modular* binding mode

The function used for computing the hydrogen bond energy in this approach is a generic function approximating hydrogen bonds in various molecular systems and unlike the interfacial hydrogen bond energy function used in approach 1 (consensus amino acids and *modular* binding mode) and 2 (consensus amino acids and *synergistic* binding mode), it is not expected to be accurate enough. Due to the large space of possible zinc fingers that this tool explores, in order to maintain feasibility, it approximates the spatial configurations of the molecules, and thus, the binding affinity with much lower resolution than approach 1 (consensus amino acids and *modular* binding mode) and 2 (consensus amino acids and *synergistic* binding mode)). While this approach shows great promise, and opens up the possibility of predicting zinc finger proteins which may be significantly different from those studied experimentally so far, there is a need to improve the resolution of the energy function to increase its accuracy. Thus, owing to the low accuracy of the tool as it stands, it has not been made available for users on our web server at the moment.

The validation process for this approach was two-fold - Firstly, the same methodology was adopted as in approach 1 (consensus amino acids and *modular* binding mode), as both the approaches are based on the same assumptions and only differ in the space of possible zinc finger proteins that they explore. It is evident from the validation that the predictions are reliable only if a minimization algorithm is incorporated into this approach. Though the predictions don’t follow the exact trends as the experimentally found K_d_ values in the case of validation with DNA sequences that have been reported to be binding well to Zif-268 in the literature, they are still much more acceptable than those for DNA sequences binding well to QNK skeleton. Since the rate of false positives is relatively high, the specificity predictions are clearly compromised in this case.

Secondly, a database consisting of 231 pairs of zinc finger (triplet)-DNA target complexes was compiled from those reported in literature. Of these, 156 gave non-zero values of interfacial hydrogen bond energy (67.5%), thus indicating that the approach was working well for this subset of the database. A higher hydrogen bond energy value (−ve assigned) corresponds to a higher ∆G value. Hence, the validation with experimental data stands in support of the tool.

Since there are multiple top scorers for each nucleotide triplet, the rate of false positives in our predictions is expected to be relatively high. This may be attributed to the fact that the mutations made by “O” to create all possibilities of the DNA-ZFP complexes resulted in PDBs which may not have incorporated the configurational changes at the rotamer level or the DNA conformational changes. One way to further refine the model and reduce the false positive rate could be to minimize the structures formed upon mutation of residues in real time. The improvement in the hydrogen bond energies shows that minimization in real time shows great promise and can be used to improve the specificity of the tool significantly (Table 4) [35–37].

**Table 4:** Validation of predictions by assuming *modular* mode of binding and mutations using all 20 amino acids (Approach 3)

Validation with DNA targets binding to QNK				Validation with DNA targets binding to Zif-268		
Target DNA 5' - 3'	K_d_ value	Predicted H-bond Energy Finger 2 (QDK) Jameison et al. 1996 [32]		Predicted H-bond Energy Finger 2 (QDK) Rebar et al. 1994 [33]	ΔG value	Hydrogen bond energy (RERRHRRER)
		Pre Minimization	Post Minimization			
GCG **TGG** GCG	16	1.3	6.7	GCG **TGG** GCG	0	32
GCG **TAG** GCG	24	1.3	5.3	GCG **TAG** GCG	0.5	30.9
GCG **GGG** GCG	142	1.3	3.4	GCG **GGG** GCG	1.3	39.3
GCG **AGG** GCG	990	1.3	1.4	GCG **AGG** GCG	1.7	44
GCG **TTG** GCG	7500	1.3	1.3	GCG **GAG** GCG	1.9	37
GCG **GAG** GCG	250000	3.7	1.17	GCG **TTG** GCG	1.9	62

A) The predicted H-bond energies after minimization follow the same trend as the K_d_ values reported in literature of various ZFP (QDK)-DNA pairs. Without the minimization, the results are inconsistent with experimental data. B) For DNA targets reported in literature binding with Zif-268 helix, the trend of predicted H-bond energies by this approach has too many outliers for the reported ΔG values for the various DNA-ZFP pairs in literature, rendering this approach unreliable

### A comparative analysis of the above three prediction approaches

As seen from the validation described above, predictions made by all the three approaches are reliable. However, because of the inherent differences in their algorithms and the assumptions of each model, a need to do a comparative analysis of the three approaches was carried out. The comparison between the predictions made by the two modes of binding and the experimental data shows that *synergistic* mode of binding, though computationally and time-intensive, clearly outperforms binding predictions made assuming the *modular* mode of binding. The *synergistic* predictions were a lot closer to mimicking the physico-chemical processes occurring at the molecular level.

**Fig. 2:**
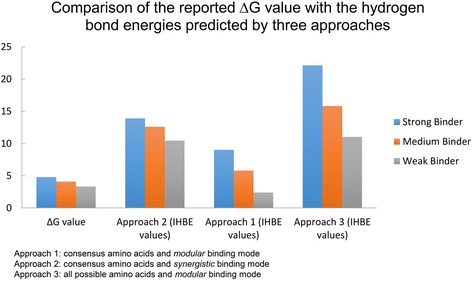
Comparative analysis and validation of all the three prediction approaches. The targets used are **a**) S1 GAGGAGGAT-RDNR RDNR QSNR **b**) M1 GAGGAAGGG-RDNR QGNR RDHR **c**) W1 GAAGAGGGT-QGNR RDNR QSHR. Binding affinity of the targets were determined based on the ∆G values owing to which they have been termed as strong, medium and weak binders as reported by *Schaal* et al. 2002 [38], where S1 is the strongest binder and W1 is the weakest. The predictions made by the three approaches developed, are in sync with the experimental data for the three binders. The trend line for ∆G has maximum resemblance to that of the predictions made by Approach 2, which assumes *synergistic* mode of binding, hence making it the most reliable of the three

From an extensive study based on DNA targets for their respective helices and their K_d_ values, it is evident that approach 2 (consensus amino acids and *synergistic* binding mode) works the best out of the three approaches (Fig. 2) [38]. As can be seen from Table 5, the trend for the predicted H-bond energies is in sync with the experimentally reported K_d_ values for all three approaches. However, the trend line for approach 2 (consensus amino acids and *synergistic* binding mode) best mimics the changes in the H-bond energy as the binding affinity (K_d_ value) is increased across sample points. Moreover, as indicated by the binding affinity predictions by all three approaches, the binding affinity increases in the order F1 < F2 < F3 (Additional file 1: Table S1). This explains why the predictions for approach 2 (consensus amino acids and *synergistic* binding mode) are the most accurate, as it takes into account this cooperation of zinc finger binding specificities (Table 5).

**Table 5:** Comparative analysis of predictions based on different modes of binding against experimental data. All 16 GNN DNA targets and their respective experimentally reported helices from Qiang et al. 2002 [17] are used to find the corresponding hydrogen bond energy for Approach (1) assuming *modular* mode of binding and the other approach assuming synergistic mode of binding (2). Evidently the energy values for Approach 2 appear more reliable than Approach 1, even though both approaches predict with reasonable efficiency

Experimentally reported data				Approach 2 : consensus amino acid and *synergistic* binding mode					Approach 1: consensus amino acid and *modular* binding mode				
Target GNN triplet (5' - 3') Qiang et al. 2002 [17]	Zinc Finger Protein Helix			H bond energy for experimental helix - target DNA complex	Helices predicted for DNA			H bond energy for predicted helix - Target DNA complex	H bond energy for experimental helix - target DNA complex	Helices predicted for DNA			H bond energy for predicted helix - Target DNA complex
	F1	F2	F3		F1	F2	F3			F1	F2	F3	
GAAGAAGAA	QNR QNR QNR			−4.05	DSGNLTT DSGNLTT DSGNLTT			−8.19	−6.62	QHR RHR DDT			−11.91
GATGATGAT	QNR TNR TNR			−3.19	HSGALTT HSGALTT HSGALTT			−5.52	−9.5	QHR RSN RHQ			−16.31
GAGGAGGAG	RNR RNR RNR			−9.27	HSGTLTN HSGTLTN HSGTLTN			−14.59	−4.8	AAK NVK ADK			−8.37
GACGACGAC	DNR DNR DNR			−8.8	RSGSLTQ RSGSLTQ RSGSLTQ			−12.69	−10.99	NHR RDK TSR			−19.22
GTAGTAGTA	QAR QAR QAR			−11.13	QSGTLTK QSGTLTK QSGTLTK			−13.76	−5.09	RDA QSR HHR			−8.17
GTTGTTGTT	TAR TAR TAR			−2.88	ASGHLTT ASGHLTT ASGHLTT			−3.99	−12.52	AAR DTT RHR			−20.47
GTGGTGGTG	RAR RAR RAR			−6.32	RSGALTQ RSGALTQ RSGALTQ			−4.29	−7.98	ASR QNQ TDR			−14.23
GTCGTCGTC	DAR DAR DAR			−7.3	HSGSLTR HSGSLTR HSGSLTR			−11.97	−10.11	THV QVT DHR			−16.58
GGAGGAGGA	QHR QHR QHR			−9.65	NSGNLTR NSGNLTR NSGNLTR			−14.91	−7.95	QVR RVR QHK			−13.14
GGTGGTGGT	QHR THR THR			−9.07	NSGVLTQ NSGVLTQ NSGVLTQ			−12.05	−11.45	AST NDR TDR			−20.40
GGGGGGGGG	RHR RHR RHR			−14.67	RSGSLTR RSGSLTR RSGSLTR			−19.44	−8.6	RHE THR NHR			−12.83
GGCGGCGGC	DHR DHR DHR			−9.77	TSGHLTQ TSGHLTQ TSGHLTQ			−10.29	−7.1	RVK DTR HDR			−12.48
GCAGCAGCA	QSR QDR QDR			−5.36	ASGTLTK ASGTLTK ASGTLTK			−6.00	−7.62	RHE DHR HHR			−12.71
GCTGCTGCT	QDR QDR QDR			−9.43	ASGALTK ASGALTK ASGALTK			−12.37	−10.71	THK QSV RSR			−18.53
GCGGCGGCG	RDR RDR RDR			−10.54	RSGNLTK RSGNLTK RSGNLTK			−10.36	−10.72	QHR RDR RSQ			−18.75
GCCGCCGCC	ETR DDR DDR			−6	NSGSLTT NSGSLTT NSGSLTT			−9.72	−8.62	ATR RHR NSK			−14.48

For Finger 3, both approach 2 (consensus amino acids and *synergistic* binding mode) and approach 1 (consensus amino acid and *modular* binding mode) yield more or less the same predictions (Additional file 2: Table S2). This could strongly be attributed to the same small pool of amino acids used to mutate key alpha helical residue positions in the ZFP-DNA complex for both the approaches. However, for finger 2, approach 3 (all possible amino acids and *modular* binding mode) functions much better. Also reported in Additional file 2: Table S1, for predictions made by approach 1 (consensus amino acid and *modular* binding mode), for 8 out of 16 GNN triplets, for finger 2, the best binding protein reported in literature was found to be within the top 20 predictions made by this approach. Similarly, for predictions made by approach 2 (consensus amino acids and *synergistic* binding mode this was true for 9 out of 16 GNN targets [39–44]. Our approach explores a large space of possible zinc finger proteins in order to identify optimal proteins to bind to a target DNA sequence, and thus, goes on to predict many proteins which have not been reported in literature. While these numbers may come across as a little disconcerting, there is a strong possibility that the predictions made by our approach are better binders than the naturally occurring ones reported in literature, as suggested by the hydrogen bond energy measure.

Additional file 3: Table S3 shows that approach 1 (consensus amino acid and *modular* binding mode) predicts position 3 for all fingers, and position 6 for F3 with greater than 90% accuracy. This hints towards the possibility, that for any zinc finger, the binding of position 3 plays a fundamental role in the overall binding affinity. A possible reason for accurate predictions of the 6th position of finger 3 could be that for most of the ZFP-DNA pairs studied for validation of this approach, this position was predominantly occupied by Arginine, which is a polar amino acid with a very high hydrogen bonding ability. Moreover, as simulation data proves, there is significant cooperation in the binding of each individual finger, such that F3 > F2 > F1, and thus, energy released upon the formation of the H-bonds with 6th position of Finger 3, is much higher than that at any other position. As we measure our binding affinity using H-bond energies released upon bond formation, the predictions for this position have a very high accuracy, given the large amount of energy released when it binds. Similarly, position −1 of F1 is also mostly dominated by amino acid R. As accurate prediction of the first position is fundamental in predicting zinc finger specificities, this goes on to increase the reliability of our approach. However, these remain to be investigated further experimentally.

### Factors affecting zinc finger binding specificities

In order to better understand the mechanisms underlying the chemistry of binding specificities in a ZFP-DNA complex, based upon the patterns revealed from the predictions of our three approaches, we have identified factors which appear to be fundamental to zinc finger binding specificity. The most important of these include the effect of zinc finger position and amino acid propensity. In light of the fact that experimentalists working on zinc fingers might benefit from these findings, we have presented them in the following section. Further, these also highlight the biological implications of the assumptions made in the three approaches.

### Amino acid preference

#### Approach 1- consensus amino acids and *modular* binding mode

**Fig. 3:**
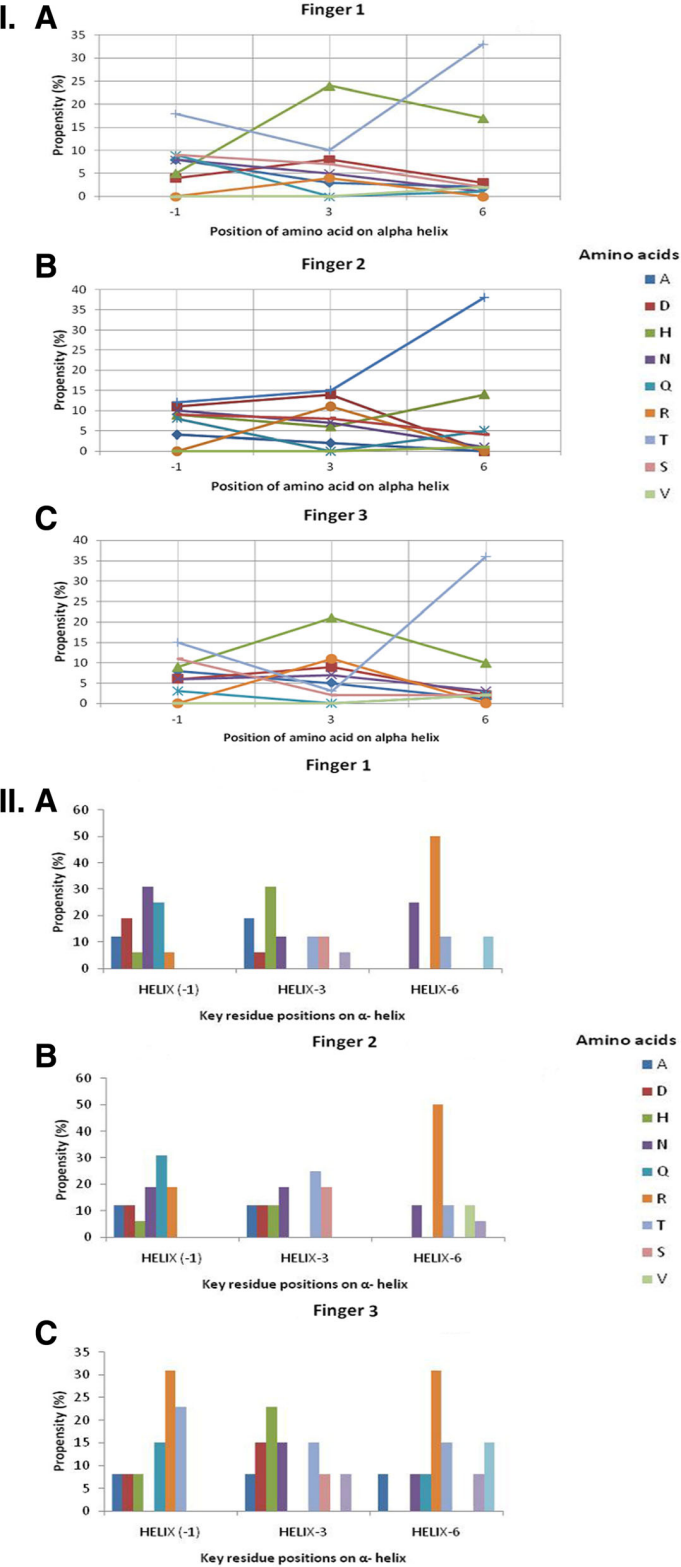
Amino acid propensity with respect to key residue positions of the ZFP helix for each finger. The propensities of all possible amino acids at position −1, +3, +6 of the top predicted ZFPs (considering all 64 codons) of a particular finger are reported. The colour coding for different amino acids are shown on the side panel. I) Amino acid propensity plots obtained by prediction Approach 1: (**Ia**) Finger 1 α-helix prefers histidine residue at 3 and arginine at −1 & 6 key residue positions. (**Ib**) Finger 2 α-helix is dominated by arginine at all positions. Aspartic acid has the same frequency as arginine at the key residue position 3. (**Ic**) Finger 3 α-helix prefers valine residue at position 3 and arginine at positions −1 & 6. II) Amino acid propensity plots obtained by prediction Approach 2: (**IIa**) Finger 1 α-helix prefers asparagine at −1, valine at 3 and arginine at 6 (**IIb**) Finger 2 α-helix is dominated by glutamine at −1, threonine at 3 and again arginine at 6 (**IIc**) Finger 3 α-helix prefers arginine at −1 & 6 and valine at 3. A high preference for arginine is seen at crucial positions like −1 and 6 of the helix, as it is a polar residue bolstering the interactions and the affinity at the interface

For finger 1, the most preferred amino acids were found to be R at position −1, K at position +3 and H at position +6 (Fig. 3). But this pattern of preferences changed when the target DNA was of the type **GN** (A/T)**N**(A/T), with the most preferred amino acids being R at position −1, H at position +3 and K/N at position +6 for Finger 1. Moreover, for the sequences of the type **GGN** (A/T), the preferred pattern was found to be R, T and T at positions −1, +3 and +6 respectively.

#### Approach 2- consensus amino acids and *synergistic* binding mode

The predictions made by this approach clearly indicate that the G nucleotide has a high affinity for binding to the arginine (R) residue on the alpha helix and that R has a high propensity for being found at positions −1 and 6 for all the three fingers. Also, if it is specifically present on the first finger, G binds with mild affinity to the amino acid sequence: RKR at −1, 3 and 6 (Fig. 3).

#### Approach 3- all possible amino acids and *modular* binding mode

Amino acid propensity from this approach appeared to be skewed as the numbers of false positives are very high and the representative data may be far from accurate (Hence data not shown). This approach shows reliability only if minimization is incorporated in the algorithm. It appears as though the process of incorporating mutations is unable to search the most optimal conformational structure for every mutant and a round of minimization would evidently optimize the mutant structure. Owing to the defect in the conformational search, the amino acids representing the data set increases the number of false positives and remains unreliable.

### Positional dependence of DNA codon

#### Approach 1- consensus amino acids and *modular* binding mode

Some DNA targets did not yield any favourable binding score, for all of the mutated helices. For finger 1, ACG, AGT and TGC, for finger 2, TAC and for finger 3, AAA, AGA, CTA, CTT, TGA and TTT did not have non-zero energy values. This could either be due to less abundance of G in the DNA target, or even, due to non-preferential binding to the mutated helices. While this remains to be investigated further, if one or more of these targets are present in the user input DNA at the particular positions mentioned above, the user is advised to select a different, preferably G-rich DNA target site. Cross checking with experimentally available data revealed that none of these codons had predictions for zinc finger helices binding to them reported in literature. However, only experimental studies can shed more light on the nucleic acid propensity and positional preferences for binding to zinc finger proteins.

DNA codons like GAC, GCC, CTT, AAC and ATG bind preferentially to different zinc fingers, depending upon their position in the 9bp DNA target sequence. The fact that the hydrogen bond energy for the same zinc finger, DNA sub-site pair can change significantly depending upon the position of the DNA sub-site proves that there is a strong effect of its position on the zinc finger binding specificity. Strong positional dependency of GCA, GAT, GGT, GAA and GCC with respect to position of the three fingers in the zinc finger mutants can be concluded. However, some consensus amino acid sequences have been found to bind to these relatively well independent of their position in the 9bp sequence and have been presented here as they might be of use to experimentalists working with zinc fingers (Table 6).

**Table 6:** Effect of DNA sub-sites (3-bp) position on the zinc finger binding specificity

Target codon^a^ 5’- 3’	Haddock scores for different positions of the codon in a 9bp target		
	F3	F2	F1
**GAT**	−134.49	−131.98	**−137.56**
**GCA**	**−153.31**	−130.95	−137.59
**GGT**	**−146.3**	−136.62	−140.01

^a^Preference of these DNA sub-sites from *Qiang* et al. 2002 [17]

GAT shows best binding when present against position Finger 1 of the zinc finger mutant, followed by F3 and F2. However, the 3bp DNA sequences GCA and GGT, show highest affinity to the respective protein sequence with respect to its position against Finger 3 respectively. These DNA mutations have been introduced in the wild type 9bp DNA sequence (5’ GCG TGG GCG 3’) binding to the prototype Zif-268. Hence the positional preference in approach 1 (consensus amino acid and modular binding mode) and 2 (consensus amino acids and synergistic binding mode) follow a similar trend as described

#### Approach 2- consensus amino acids and *synergistic* binding mode

It can be inferred from the prediction results that GAT (NNN-type) DNA sequence shows best binding with respect to its position against Finger 1 of the zinc finger mutant followed by Finger 3 and Finger 2 position, as indicated by the significantly lower HADDOCK scores. Moreover, the 3bp sequences GCA and GGT show the highest affinity to their corresponding protein targets when present at a position against Finger 3 of the zinc finger mutant (Table 6).

#### Approach 3- all possible amino acids and *modular* binding mode

DNA targets like GGG, GCG, GTG, GAC show higher binding affinity with respect to its position against the fingers of the zinc finger mutant in the order: F3 > F2 > F1 for experimental as well as our predictions from approach 3 (Table 7).

**Table 7:** Effect of DNA sub-sites (3-bp) position on the zinc finger binding specificity

Experimental					Approach 3
DNA Target sequence 5’ – 3’			DNA sub-site position w.r.t to F1, F2, F3	Corresponding Helix	IHBE score for experimental DNA target and corresponding Helix
GGG	Jamieson et al. 1996 [32]	GGG	1	RER	0.0
	Segal et al. 1999 [45]	GGG	2	RHR	39.3
	Van et al. 2004 [46]	**GGG**	**3**	**RHR**	**50.63**
GCG	Hurt et al. 2003 [44]	**GCG**	**1**	**RER**	**30.9**
	Van et al. 2004 [46]	GCAGCGGAG	2	RER	21.64
	Kim et al. 2010 [43]	GCGGGGGCG	3	RER	28.6
GTG	Jamieson et al. 1996 (32)	GTG	1	RER	1.35
	Hurt et al. 2003 [44]	**GCGGTGGCG**	**2**	**RER**	**19.59**
	Van et al. 2004 [46]	GTGGACGAA	3	RAR	5.5
GAC	Rebar et al. 2002 [39]	GGGGGTGAC	1	DNR	2.04
	Holmes et al. 2005 [40]	GTGGACGAA	2	DNR	4.033
	**Bae et al. 2003** [41]	**GAC**	**3**	**CNR**	**7.23**

In Approach 3, GGG and GAC, show highest affinity with the respective protein sequence when present at a position against Finger 3, whereas GCG and GTG show high affinity for corresponding helix reported from literature for its position against Finger 1 and Finger 2 respectively

## Conclusions

The three approaches presented here differ primarily in two aspects - Firstly, the mode of binding assumed and secondly, the space of Zif-268 mutants analysed for finding potential ZFPs for any DNA target. While the predictions made by all three approaches show coherence with the data reported in literature, each has its strengths and its pitfalls.

Approach 1 makes predictions about ZFP-DNA binding specificities assuming *modular* binding and searches for optimal zinc fingers using a small pool of amino acids shows great promise and is the most reliable of the three approaches. However, approach 2, which introduces mutations in the Zif-268 template protein from the same pool of amino acids as approach 1, assumes *synergistic* mode of binding between ZFP-DNA, and thus incorporates the co-operative effect of their binding. This allows it to best mimic the physico-chemical interactions at the molecular level. But due to the large number of possible complexes in this approach, it is computationally intensive. Both Approach 1 and 2 base their predictions on a score calculated using the interfacial hydrogen bond energy. Our results show that this score can be safely used as an indirect indicator for binding affinity. Both approaches have been made available for users on an easy to use web server, which helps users identify optimal zinc finger proteins for any 9bp DNA target sequence.

Approach 3, which assumes *modular* binding like approach 1 searches for potential zinc finger proteins by introducing all possible mutations in the Zif-268 skeleton. This helps reduce the time required for the large number of computations by relying on a different methodology for the introduction of mutations, and for the calculation of the binding affinity based score. Due to the inherent weaknesses in these steps, approach 3 is plagued with a much higher false positive rate, which makes it infeasible as it stands right now. Initial results show that real time minimization of the structures after the introduction of mutations might help improve the accuracy of the tool significantly, however, this remains to be investigated further. In light of these facts, this approach has not been made available for users on our web server at the moment.

The strength of these approaches lie in that they are derived from physico-chemical parameters, and thus, independent of experimental data. This subverts the need to update the approaches every time new data is produced by experimental research. Moreover, they are free of any bias that might be introduced by limited or uneven research in the field. However, each of these approaches is dependent on a certain binding model and labours under assumptions that were incorporated to optimize computation. A better understanding of the mechanism of the ZFP-DNA complex formation will go a long way in improving such techniques and can be expected to have an important role to play in designing tools for targeted genome editing.

## Additional files

Additional file 1: Table S1. Detailed analysis of predictions against experimental data for Approaches 1 and 3 for all 16 GNN triplets for different finger (1, 2 or 3 positions). Experimental data involves target DNA sequence which binds to its respective helix3 on the ZFP at various positions (finger 1, finger 2 & finger 3) with the corresponding K_d_ values for determining experimental affinity between DNA and its respective ZFP. Approach 3 (all possible amino acids and *modular* binding mode) gives its top helix for the experimental DNA target with its rank and score respectively. Approach 1 (consensus amino acid and *modular* binding mode) gives its top helix for the experimental DNA target with its rank and IHBE score respectively. Approach 1 predicts zinc finger helices for experimental DNA targets accurately for Finger 3 followed by Finger 1, whereas Approach 3 does so for Finger 2. (DOCX 56 kb)
Additional file 2: Table S2. Position wise (-1, 3, 6 of the α-helix) analysis for experimental against Approaches 1 and 3 for all 16 GNN triplets for different finger (F1, F2 or F3 positions). Experimental data involves GNN-type DNA sequence that binds to its respective amino acids at -1,3 & 6 positions on each α-helix on the ZFP at various fingers (finger 1, finger 2 & finger 3). Approach 1 uses a consensus pool of amino acids derived from literature for mutating the cardinal helix residues for predictions. Approach 3 explains about the algorithm used by ZIF predict IHBE to predict helices for the target DNA Sequence where the key mutations in the ZFP helix involve all 20 amino acids randomized at all the three positions (-1,3 and 6 on each finger-helix). Position 3 for all fingers almost predict with >90% accuracy for Approach 1. Further position 6 of F3 also predicted with >90% accuracy in both Approach 1 & 3. (DOCX 30 kb)
Additional file 3: Table S3. Checking top predictions (top 50) to establish relationship between Approach 2 (consensus amino acids and synergistic binding mode) and Approach 1 (consensus amino acid and modular binding mode) prediction for all 16 GNN targets. Our Approach 2 predictions for Finger 3 coincide with the Approach 1 predictions. (DOCX 23 kb)
