# Supplementary material for: Exploiting the recognition code for elucidating the mechanism of zinc finger protein-DNA interactions
Source: BMC Genomics. 2016 Dec 22;17(Suppl 13):1037. doi: 10.1186/s12864-016-3324-8 (PMC5260074; doi:10.1186/s12864-016-3324-8)
Supplement: Additional file 1: Table S1. — Detailed analysis of predictions against experimental data for Approaches 1 and 3 for all 16 GNN triplets for different finger (1, 2 or 3 positions). Experimental data involves target DNA sequence which binds to its respective helix3 on the ZFP at various positions (finger 1, finger 2 & finger 3) with the corresponding Kd values for determining experimental affinity between DNA and its respective ZFP. Approach 3 (all possible amino acids and modular binding mode) gives its top helix for the experimental DNA target with its rank and score respectively. Approach 1 (consensus amino acid and modular binding mode) gives its top helix for the experimental DNA target with its rank and IHBE score respectively. Approach 1 predicts zinc finger helices for experimental DNA targets accurately for Finger 3 followed by Finger 1, whereas Approach 3 does so for Finger 2. (DOCX 56 kb) [file 12864_2016_3324_MOESM1_ESM.docx]

### Additional file 1 – Detailed analysis of predictions against experimental data for Approaches 1 and 3 for all 16 GNN triplets for different finger (1, 2 or 3 positions)

Experimental data involves target DNA sequence which binds to its respective helix3 on the ZFP at various positions (finger 1, finger 2 & finger 3) with the corresponding K_d_ values for determining experimental affinity between DNA and its respective ZFP. Approach 3 (all possible amino acids and *modular* binding mode) gives its top helix for the experimental DNA target with its rank and score respectively. Approach 1 (consensus amino acid and *modular* binding mode) gives its top helix for the experimental DNA target with its rank and IHBE score respectively. Approach 1 predicts zinc finger helices for experimental DNA targets accurately for Finger 3 followed by Finger 1, whereas Approach 3 does so for Finger 2.

| **Experimental** | | | | **Approach 3** | | | **Approach 1** | | |
| --- | --- | --- | --- | --- | --- | --- | --- | --- | --- |
| **Target sequence** | **position** | **helix** | **K_d_** | **Top helix** | **Rank/percentile** | **score** | **helix** | **rank** | **score** |
| GGG[[32](#_ENREF_32)] | 1 | RER | 5.6 | RCR | 54.4 | 0.0 | RHE | 7 | 1.8 |
| GGG | 1 | RHK | .055 |  | 80 | 36.2 |  |  |  |
| GGG[[13](#_ENREF_13)] | 1 |  |  |  |  |  |  |  |  |
| GGG[[45](#_ENREF_45)] | 2 | RHR | .4 | RRS | 89.9 | 39.3 | THR |  |  |
| GGG[[14](#_ENREF_14)] | 2 | RKR | 6 |  | 99.73 | 61.4 |  |  |  |
| GCGGGGGCG | 2 | RHR | 1.3 |  |  |  |  |  |  |
| GGG | 2 | RKR | 6 |  | 95 | 56.2 |  |  |  |
| GGG[[45](#_ENREF_45)] | 3 | RHR | 0.01 | RYR |  |  | NHR | 4 | 3.2 |
| GGG | 3 | RHR | <0.01 |  |  |  |  |  |  |
| GGG[[46](#_ENREF_46)] | 3 | RHR | 0.001 |  | 94.75 | 50.63 |  |  |  |
| GGG[[36](#_ENREF_36)] | 3 | KHA | 0.055 |  | 74.75 | 7.2 |  |  |  |
| GGG[[32](#_ENREF_32)] | 3 | RKR | 81 |  |  |  |  |  |  |

GGG

GCG

| **Experimental** | | | | **Approach 3** | | | **Approach 1** | | |
| --- | --- | --- | --- | --- | --- | --- | --- | --- | --- |
| **Target sequence** | **position** | **helix** | **K_d_** | **Top helix** | **Rank/percentile** | **score** | **helix** | **rank** | **score** |
| GCG[[32](#_ENREF_32)] | 1 | RER |  | RYA | 80 | 30.9 | QHR |  |  |
| GCG [[44](#_ENREF_44)] | 1 | RER | 0.5 |  |  |  |  |  |  |
| GCG [[37](#_ENREF_37)] | 2 | RDR | 0.17 | RRS | 88.5 | 19.06 | RDR | 1 | 5.5 |
| GCGGCGGCG [[45](#_ENREF_45)] | 2 | RDR | 9 |  |  |  |  |  |  |
| GCAGCGGAG [[38](#_ENREF_38)] | 2 | RER | 0.031 |  | 89.25 | 21.64 |  |  |  |
| GGGGCGGGG[[2](#_ENREF_2)] | 2 | RER | 0.055 |  |  |  |  |  |  |
| GCG[[33](#_ENREF_33)] | 2 | RDR | 9 |  |  |  |  |  |  |
| GCGGGGGCG[[43](#_ENREF_43)] | 3 | RER | 0.057 | RYR | 94.5 | 18.6 | RSQ | 29 | .23 |

GTG

| **Experimental** | | | | **Approach 3** | | | **Approach 1** | | |
| --- | --- | --- | --- | --- | --- | --- | --- | --- | --- |
| **Target sequence** | **position** | **helix** | **K_d_** | **Top helix** | **Rank/percentile** | **score** | **helix** | **rank** | **Score** |
| GTG [[32](#_ENREF_32)] | 1 | RER | 3.4 | RYH | 80 | 1.35 | ASR |  |  |
| GTG[[2](#_ENREF_2)] | 2 | RER | 15 | RHS |  |  |  |  |  |
| GTG[[44](#_ENREF_44)] | 2 | RSR | 3 |  | 83 | 19.6 | QNQ | 3 | 2.3 |
| GCGGTGGCG[[43](#_ENREF_43)] | 2 | RER | 15 |  | 83.6 | 19.59 |  |  |  |
| GTGGACGAA[[33](#_ENREF_33)] | 3 | RAR | .003 | RYR | 69.25 | 5.5 | TDR | 77 | 0.74 |
| GTGGTAGAC [[45](#_ENREF_45)] | 3 | RAR | 0.690 |  |  |  |  |  |  |

GGC

| **Experimental** | | | | **Approach 3** | | | **Approach 1** | | |
| --- | --- | --- | --- | --- | --- | --- | --- | --- | --- |
| **Target sequence** | **position** | **helix** | **K_d_** | **Top helix** | **Rank/percentile** | **score** | **helix** | **rank** | **Score** |
| GGC[[32](#_ENREF_32)] | 2 | DHR | 40 | RRS | 89.5 | 19.6 | DTR | 21 | 0.3 |
| GCGGGCGCG[[33](#_ENREF_33)] | 2 | DHR | 40 |  |  |  |  |  |  |
| GCTGGGGGC [[46](#_ENREF_46)] | 3 | DHR | <0.01 | RYR | 95 | 56.21 | HDR | 87 | 0.09 |

GGA

| **Experimental** | | | | **Approach 3** | | | **Approach 1** | | |
| --- | --- | --- | --- | --- | --- | --- | --- | --- | --- |
| **Target sequence** | **position** | **helix** | **K_d_** | **Top helix** | **Rank/percentile** | **score** | **helix** | **rank** | **Score** |
| GGA[[32](#_ENREF_32)] | 1 | QHR | 7.4 | RCR | 80 | 36.2 | QHR | 5 | .8 |
| GCGGGAGCG[[33](#_ENREF_33)] | 2 | QHR | 3 | RRS | 88.7 | 19.6 |  |  |  |
| GCGGGAGCG[[44](#_ENREF_44)] | 2 | RHR |  |  | 89.9 | 34.8 | RVR | 121 | .004 |
| GGA [[2](#_ENREF_2)] | 2 | QHR | 3 |  |  |  |  |  |  |
| GGA [[37](#_ENREF_37)] | 3 | QHR |  | RYR | 95 | 56.2 | QHR | 1 | 2.7 |

GGT

| **Experimental** | | | | **Approach 3** | | | **Approach 1** | | |
| --- | --- | --- | --- | --- | --- | --- | --- | --- | --- |
| **Target sequence** | **position** | **helix** | **K_d_** | **Top helix** | **Rank/percentile** | **score** | **helix** | **rank** | **Score** |
| GAGGAAGGT[[2](#_ENREF_2)] | 1 | THR | <0.06 | RCY | 80 | 36.2 | AST | 146 | 0.8 |
| GAAGAGGGT[[32](#_ENREF_32)] | 1 | QHR | .0036 |  | 81 | 36.3 |  | 10 | 4.5 |
| AGTGGTGAG [[43](#_ENREF_43)] | 2 | QHR | 0.340 | KRS | 89.2 | 19.6 | NDR | 112 | 0.6 |
| GGT[[33](#_ENREF_33)] | 2 | THR | 15 |  | 89.3 | 19.61 |  |  |  |
| GGGGGTGAC [[44](#_ENREF_44)] | 2 | THR | <0.01 |  |  |  |  |  |  |
| GGT[6] | 2 | THR | 15 |  |  |  |  |  |  |
|  |  |  |  |  |  |  | TDR |  |  |

GCC

| **Experimental** | | | | **Approach 3** | | | **Approach 1** | | |
| --- | --- | --- | --- | --- | --- | --- | --- | --- | --- |
| **Target sequence** | **position** | **helix** | **K_d_** | **Top helix** | **Rank/percentile** | **score** | **helix** | **rank** | **Score** |
| GCGTGGGCC[[33](#_ENREF_33)] | 1 | RER | 3 | RCR | 80 | 30.98 | ATR |  |  |
| GCC[[32](#_ENREF_32)] | 2 | DDR | 80 |  | 61.75 | 1.33 |  | 12 | 0.7 |
|  | 2 | DNR | 90 |  | 79.25 | 4.03 |  |  |  |
|  | 2 | DAR | (Low binding) | RRS | 56.99 | 0 | RHR |  |  |
| GCC[[2](#_ENREF_2)] | 2 | DDR |  |  |  |  |  |  |  |
| GCC [[43](#_ENREF_43)] | 3 | DCR | 0.22 |  | 64.5 | 5.5 |  |  |  |
| GCCTGATGG[8] | 3 | DDR | 1.3 | RYR | 89.5 | 16.3 | NSR |  |  |

GGT

| **Experimental** | | | | **Approach 3** | | | **Approach 1** | | |
| --- | --- | --- | --- | --- | --- | --- | --- | --- | --- |
| **Target sequence** | **position** | **helix** | **K_d_** | **Top helix** | **Rank/percentile** | **score** | **helix** | **rank** | **Score** |
| GAGGAAGGT[[2](#_ENREF_2)] | 1 | THR | <0.06 | RCY | 80 | 36.2 | AST | 146 | 0.8 |
| GAAGAGGGT[[32](#_ENREF_32)] | 1 | QHR | .0036 |  | 81 | 36.3 |  | 10 | 4.5 |
| AGTGGTGAG [[43](#_ENREF_43)] | 2 | QHR | 0.340 | KRS | 89.2 | 19.6 | NDR | 112 | 0.6 |
| GGT[[33](#_ENREF_33)] | 2 | THR | 15 |  | 89.3 | 19.61 |  |  |  |
| GGGGGTGAC [[44](#_ENREF_44)] | 2 | THR | <0.01 |  |  |  |  |  |  |
| GGT[6] | 2 | THR | 15 |  |  |  | TDR |  |  |

GCC

| **Experimental** | | | | **Approach 3** | | | **Approach 1** | | |
| --- | --- | --- | --- | --- | --- | --- | --- | --- | --- |
| **Target sequence** | **position** | **helix** | **K_d_** | **Top helix** | **Rank/percentile** | **score** | **helix** | **rank** | **Score** |
| GCGTGGGCC([33](#_ENREF_33)) | 1 | RER | 3 | RCR | 80 | 30.98 | ATR |  |  |
| GCC([32](#_ENREF_32)) | 2 | DDR | 80 |  | 61.75 | 1.33 |  | 12 | 0.7 |
|  | 2 | DNR | 90 |  | 79.25 | 4.03 |  |  |  |
|  | 2 | DAR | (Low binding) | RRS | 56.99 | 0 | RHR |  |  |
| GCC([2](#_ENREF_2)) | 2 | DDR |  |  |  |  |  |  |  |
| GCC ([43](#_ENREF_43)) | 3 | DCR | 0.22 |  | 64.5 | 5.5 |  |  |  |
| GCCTGATGG[8] | 3 | DDR | 1.3 | RYR | 89.5 | 16.3 | NSR |  |  |

GAA

| **Experimental** | | | | **Approach 3** | | | **Approach 1** | | |
| --- | --- | --- | --- | --- | --- | --- | --- | --- | --- |
| **Target sequence** | **position** | **helix** | **K_d_** | **Top helix** | **Rank/percentile** | **score** | **helix** | **rank** | **Score** |
| TTGGAAGAA([32](#_ENREF_32)) | 1,2 | QNR, QNR | 0.30 | RWR | 70 | 2.04 | QHR | 32 | 0.15 |
| GTGGAGGAA[6] | 1 | QNR | 0.03 |  |  |  |  |  |  |
| GAA([45](#_ENREF_45)) | 2 | QNR | 0.5 | RKS | 70.2 | 4.03 | RHR |  |  |
| GGGGAAAGG[7] | 2 | QNR | 0.0036 |  |  |  |  |  |  |
| GAGGAAGGG([33](#_ENREF_33)) | 2 | QNR | 0.001 |  |  |  |  |  |  |
| GAA[9] | 2 | QNR | 0.069 |  |  |  |  |  |  |
| GAGGAAGGT([44](#_ENREF_44)) | 2 | QNR | 0.06 |  |  |  |  |  |  |

GTT

| **Experimental** | | | | **Approach 3** | | | **Approach 1** | | |
| --- | --- | --- | --- | --- | --- | --- | --- | --- | --- |
| **Target sequence** | **position** | **helix** | **K_d_** | **Top helix** | **Rank/percentile** | **score** | **helix** | **rank** | **Score** |
| GCGTGGGTT([32](#_ENREF_32)) | 1 | RER | - | RYR | 80 | 1.3 | AAR |  |  |
| GTT([45](#_ENREF_45)) | 2 | TSR | 3 |  | 51.29 |  |  | 14 | 3.4 |
| GTT([41](#_ENREF_41)) | 2 | HSR | 0.43 | RHS |  |  | DTT | 87 | 2.18 |
| GTT([42](#_ENREF_42)) | 2 | TSR | 5 |  |  |  |  |  |  |
|  |  |  |  | RYR |  | RHR |  |  |  |

GAT

| **Experimental** | | | | **Approach 3** | | | **Approach 1** | | |
| --- | --- | --- | --- | --- | --- | --- | --- | --- | --- |
| **Target sequence** | **position** | **helix** | **K_d_** | **Top helix** | **Rank/percentile** | **score** | **helix** | **rank** | **Score** |
| GAGGAGGAT([46](#_ENREF_46)) | 1 | QNR | .0003 | RWR | 70 | 0.0 | RDA | 17 | 1.33 |
| GAT ([45](#_ENREF_45)) | 2 | TNR | 3 |  |  |  |  |  |  |
| GGGGATGAC([43](#_ENREF_43)) | 2 | TNR | 81 | RKS | 70.28 | 4.03 | QSR | 79 | 0.14 |
|  |  |  |  | RHR |  |  | RHQ |  |  |

GTA

| **Experimental** | | | | **Approach 3** | | | **Approach 1** | | |
| --- | --- | --- | --- | --- | --- | --- | --- | --- | --- |
| **Target sequence** | **position** | **helix** | **K_d_** | **Top helix** | **Rank/percentile** | **score** | **helix** | **rank** | **Score** |
|  |  |  |  | RYR | 51.2 | 0.0 | RDA |  |  |
| GAT([45](#_ENREF_45)) | 2 | QSR | 25 |  |  |  |  |  |  |
| GTGGTAGAC([44](#_ENREF_44)) | 2 | QSR | 0.69 | RHS |  |  | QSR | 1 | 2.5 |
|  |  |  |  | RYR |  |  |  |  |  |

GAC

| **Experimental** | | | | **Approach 3** | | | **Approach 1** | | |
| --- | --- | --- | --- | --- | --- | --- | --- | --- | --- |
| **Target sequence** | **position** | **helix** | **K_d_** | **Top helix** | **Rank/percentile** | **score** | **helix** | **rank** | **Score** |
| GTGGTAGAC([40](#_ENREF_40)) | 1 | DNR | 0.69 | RWR | 70 | 2.04 | NHR |  |  |
| GGGGATGAC([43](#_ENREF_43)) | 1 | DNR | 81 |  |  |  | DHR |  |  |
| GGGGGTGAC([39](#_ENREF_39)) | 1 | DNR | <0.01 |  |  |  |  |  |  |
| GTGGACGAA([40](#_ENREF_40)) | 2 | DNR | 0.003 | RKR | 74.01 | 4.033 | TSN | 120 | 1.33 |
| GAC([45](#_ENREF_45)) | 2 | DNR | 3 |  |  |  |  |  |  |
| GAC([41](#_ENREF_41)) | 3 | CNR | 0.13 | RHR | 79.75 | 7.23 | TSR |  |  |

GCA

| **Experimental** | | | | **Approach 3** | | | **Approach 1** | | |
| --- | --- | --- | --- | --- | --- | --- | --- | --- | --- |
| **Target sequence** | **position** | **helix** | **K_d_** | **Top helix** | **Rank/percentile** | **score** | **helix** | **rank** | **Score** |
| GCGTGGGCA([32](#_ENREF_32)) | 1 | RER | 2.4 | RYR | 80 | 30.98 | RHE |  |  |
| GCA([45](#_ENREF_45)) | 2 | QDR | 2 | RRS | 55.57 | 1.33 | DHR | 16 | 0.5 |
| GCA([42](#_ENREF_42)) | 2 | QDR |  |  |  |  |  |  |  |
| GCAGCGGAC([39](#_ENREF_39)) | 3 | QSR | 0.031 | RYR | 64.5 | 5.5 | HHR |  |  |

GTC

| **Experimental** | | | | **Approach 3** | | | **Approach 1** | | |
| --- | --- | --- | --- | --- | --- | --- | --- | --- | --- |
| **Target sequence** | **position** | **helix** | **K_d_** | **Top helix** | **Rank/percentile** | **score** | **helix** | **rank** | **Score** |
| GTC([42](#_ENREF_42)) | 2 | DAR | 40 | RHS | 54.1 | 0.0 |  |  |  |
| GTC([45](#_ENREF_45)) | 2 | DAR |  |  |  |  | QVT |  |  |
|  |  |  |  | RYR |  |  | DHR |  |  |

GCT

| **Experimental** | | | | **Approach 3** | | | **Approach 1** | | |
| --- | --- | --- | --- | --- | --- | --- | --- | --- | --- |
| **Target sequence** | **position** | **helix** | **K_d_** | **Top helix** | **Rank/percentile** | **score** | **helix** | **rank** | **score** |
| GCGTGGGCT([32](#_ENREF_32)) | 1 | RER | 3.7 | RYR | 80 | 30.98 | THR |  |  |
| GCT([45](#_ENREF_45)) | 2 | TER | 65 |  | 70.77 | 3.3 |  |  |  |
| GCT([45](#_ENREF_45)) | 2 | QDR | 10 | RRS | 58.6 | 1.3 | QSV | 3 | 4.6 |
| GCT([42](#_ENREF_42)) | 2 | TER |  |  |  |  |  |  |  |
| GCTGGGGGC([39](#_ENREF_39)) | 3 | QDR | <0.01 | RYR | 89.5 | 16.3 | RSR | 50 | 1.01 |
