# Supplementary material for: Exploiting the recognition code for elucidating the mechanism of zinc finger protein-DNA interactions
Source: BMC Genomics. 2016 Dec 22;17(Suppl 13):1037. doi: 10.1186/s12864-016-3324-8 (PMC5260074; doi:10.1186/s12864-016-3324-8)
Supplement: Additional file 2: Table S2. — Position wise (-1, 3, 6 of the α-helix) analysis for experimental against Approaches 1 and 3 for all 16 GNN triplets for different finger (F1, F2 or F3 positions). Experimental data involves GNN-type DNA sequence that binds to its respective amino acids at -1,3 & 6 positions on each α-helix on the ZFP at various fingers (finger 1, finger 2 & finger 3). Approach 1 uses a consensus pool of amino acids derived from literature for mutating the cardinal helix residues for predictions. Approach 3 explains about the algorithm used by ZIF predict IHBE to predict helices for the target DNA Sequence where the key mutations in the ZFP helix involve all 20 amino acids randomized at all the three positions (-1,3 and 6 on each finger-helix). Position 3 for all fingers almost predict with >90% accuracy for Approach 1. Further position 6 of F3 also predicted with >90% accuracy in both Approach 1 & 3. (DOCX 30 kb) [file 12864_2016_3324_MOESM2_ESM.docx]

### Additional file 2 – Position wise (-1, 3, 6 of the α-helix) analysis for experimental against Approaches 1 and 3 for all 16 GNN triplets for different finger (F1, F2 or F3 positions)

Experimental data involves GNN-type DNA sequence that binds to its respective amino acids at -1,3 & 6 positions on each α-helix on the ZFP at various fingers (finger 1, finger 2 & finger 3).. Approach 1 uses a consensus pool of amino acids derived from literature for mutating the cardinal helix residues for predictions. Approach 3 explains about the algorithm used by ZIF predict IHBE to predict helices for the target DNA Sequence where the key mutations in the ZFP helix involve all 20 amino acids randomized at all the three positions (-1,3 and 6 on each finger-helix). Position 3 for all fingers almost predict with >90%accuracy for Approach 1. Further position 6 of F3 also predicted with >90% accuracy in both Approach 1 & 3.

| **GNN** | **Experiment type** | **Finger 1** | | | **Finger 2** | | | **Finger 3** | | |
| --- | --- | --- | --- | --- | --- | --- | --- | --- | --- | --- |
| **GGG** |  | -1 | 3 | 6 | -1 | 3 | 6 | -1 | 3 | 6 |
|  | 1.Exp | R | E | R | R | H | R | R | H | R |
|  |  | R | H | K | R | K | R | R | K | R |
|  |  |  |  |  |  |  |  | K | H | A |
|  | 2. Predictions from throughout | | | | | | | | | |
|  | a. Approach 1 | R | H | E | T | H | R | N | H | R |
|  | b. Approach 3 | R | C | R | R | R | S | R | Y | R |

| **GNN** | **Experiment type** | **Finger 1** | | | **Finger 2** | | | **Finger 3** | | |
| --- | --- | --- | --- | --- | --- | --- | --- | --- | --- | --- |
| **GCG** |  | -1 | 3 | 6 | -1 | 3 | 6 | -1 | 3 | 6 |
|  | 1.Exp | R | E | R | R | D | R | R | E | R |
|  |  |  |  |  | R | E | R | R | D | R |
|  | 2. Predictions from throughout | | | | | | | | | |
|  | a. Approach 1 | Q | H | R | R | D | R | R | S | Q |
|  | b. Approach 3 | R | Y | A | R | R | S | R | Y | R |

| **GNN** | **Experiment type** | **Finger 1** | | | **Finger 2** | | | **Finger 3** | | |
| --- | --- | --- | --- | --- | --- | --- | --- | --- | --- | --- |
| **GTG** |  | -1 | 3 | 6 | -1 | 3 | 6 | -1 | 3 | 6 |
|  | 1.Exp | R | E | R | R | S | R | R | A | R |
|  |  |  |  |  | R | E | R |  |  |  |
|  | 2. Predictions from throughout | | | | | | | | | |
|  | a. Approach 1 | A | S | R | Q | N | Q | T | D | R |
|  | b. Approach 3 | R | Y | H | R | H | S | R | Y | R |

| **GNN** | **Experiment type** | **Finger 1** | | | **Finger 2** | | | **Finger 3** | | |
| --- | --- | --- | --- | --- | --- | --- | --- | --- | --- | --- |
|  |  | -1 | 3 | 6 | -1 | 3 | 6 | -1 | 3 | 6 |
| **GGC** | 1.Exp |  |  |  | D | H | R | D | H | R |
|  | 2. Predictions from throughout | | | | | | | | | |
|  | a. Approach 1 |  |  |  | D | T | R | H | D | R |
|  | b. Approach 3 |  |  |  | R | R | S | R | Y | R |

| **GNN** | **Experiment type** | **Finger 1** | | | **Finger 2** | | | **Finger 3** | | |
| --- | --- | --- | --- | --- | --- | --- | --- | --- | --- | --- |
| **GGA** |  | -1 | 3 | 6 | -1 | 3 | 6 | -1 | 3 | 6 |
|  | 1.Exp | Q | H | R | Q | H | R | Q | H | R |
|  |  |  |  |  | R | H | R |  |  |  |
|  | 2. Predictions from throughout | | | | | | | | | |
|  | a. Approach 1 | Q | H | R | R | V | R | Q | H | R |
|  | b. Approach 3 | R | C | R | R | R | S | R | Y | R |

| **GNN** | **Experiment type** | **Finger 1** | | | **Finger 2** | | | **Finger 3** | | |
| --- | --- | --- | --- | --- | --- | --- | --- | --- | --- | --- |
| **GGT** |  | -1 | 3 | 6 | -1 | 3 | 6 | -1 | 3 | 6 |
|  | 1.Exp | T | H | R | Q | H | R | T | H | R |
|  |  | Q | H | R | T | H | R |  |  |  |
|  |  |  |  | T | H | R |  |  |  |  |
|  | 2. Predictions from throughout | | | | | | | | | |
|  | a. Approach 1 | A | S | T | N | D | R | T | D | R |
|  | b. Approach 3 | R | C | Y | K | R | S |  |  |  |

| **GNN** | **Experiment type** | **Finger 1** | | | **Finger 2** | | | **Finger 3** | | |
| --- | --- | --- | --- | --- | --- | --- | --- | --- | --- | --- |
| **GCC** |  | -1 | 3 | 6 | -1 | 3 | 6 | -1 | 3 | 6 |
|  | 1.Exp | R | E | R | D | D | R | D | C | R |
|  |  |  |  |  | D | N | R | D | D | R |
|  |  |  |  |  | D | A | R |  |  |  |
|  | 2. Predictions from throughout | | | | | | | | | |
|  | a. Approach 1 | A | T | R | R | H | R | N | S | R |
|  | b. Approach 3 | R | C | R | R | R | S | R | Y | R |

| **GNN** | **Experiment type** | **Finger 1** | | | **Finger 2** | | | **Finger 3** | | |
| --- | --- | --- | --- | --- | --- | --- | --- | --- | --- | --- |
|  |  | -1 | 3 | 6 | -1 | 3 | 6 | -1 | 3 | 6 |
| GAA | 1.Exp | Q | N | R | Q | N | R | Q | N | R |
|  | 2. Predictions from throughout | | | | | | | | | |
|  | a. Approach 1 | Q | H | R | R | H | R |  |  |  |
|  | b. Approach 3 | R | W | R | R | K | S |  |  |  |

| **GNN** | **Experiment type** | **Finger 1** | | | **Finger 2** | | | **Finger 3** | | |
| --- | --- | --- | --- | --- | --- | --- | --- | --- | --- | --- |
|  |  | -1 | 3 | 6 | -1 | 3 | 6 | -1 | 3 | 6 |
| GTT | 1.Exp | R | E | R | T | S | R |  |  |  |
|  |  |  |  |  | H | S | R |  |  |  |
|  | 2. Predictions from throughout | | | | | | | | | |
|  | a. Approach 1 | A | A | R | D | T | T | R | H | R |
|  | b. Approach 3 | R | Y | R | R | H | S | R | Y | R |

| **GNN** | **Experiment type** | **Finger 1** | | | **Finger 2** | | | **Finger 3** | | |
| --- | --- | --- | --- | --- | --- | --- | --- | --- | --- | --- |
| **GAT** |  | -1 | 3 | 6 | -1 | 3 | 6 | -1 | 3 | 6 |
|  | 1.Exp | Q | N | R | T | N | R |  |  |  |
|  | 2. Predictions from throughout | | | | | | | | | |
|  | a. Approach 1 | R | D | A | Q | S | R | R | H | Q |
|  | b. Approach 3 | R | W | R | R | K | S | R | H | R |

| **GNN** | **Experiment type** | **Finger 1** | | | **Finger 2** | | | **Finger 3** | | |
| --- | --- | --- | --- | --- | --- | --- | --- | --- | --- | --- |
| **GTA** |  | -1 | 3 | 6 | -1 | 3 | 6 | -1 | 3 | 6 |
|  | 1.Exp |  |  |  | Q | S | R |  |  |  |
|  | 2. Predictions from throughout | | | | | | | | | |
|  | a. Approach 1 | R | D | A | Q | S | R |  |  |  |
|  | b. Approach 3 | R | Y | R | R | H | S | R | Y | R |

| **GNN** | **Experiment type** | **Finger 1** | | | **Finger 2** | | | **Finger 3** | | |
| --- | --- | --- | --- | --- | --- | --- | --- | --- | --- | --- |
| **GAC** |  | -1 | 3 | 6 | -1 | 3 | 6 | -1 | 3 | 6 |
|  | 1.Exp | D | N | R | D | N | R | D | N | R |
|  |  |  |  |  |  |  |  | C | N | R |
|  | 2. Predictions from throughout | | | | | | | | | |
|  | a. Approach 1 | N | H | R | T | S | N | T | S | R |
|  | b. Approach 3 | R | W | R | R | K | R | R | H | R |

| **GNN** | **Experiment type** | **Finger 1** | | | **Finger 2** | | | **Finger 3** | | |
| --- | --- | --- | --- | --- | --- | --- | --- | --- | --- | --- |
| **GCA** |  | -1 | 3 | 6 | -1 | 3 | 6 | -1 | 3 | 6 |
|  | 1.Exp | R | E | R | Q | D | R | Q | S | R |
|  | 2. Predictions from throughout | | | | | | | | | |
|  | a.Approach 1 | R | H | E | D | H | R | H | H | R |
|  | b.Approach 3 | R | Y | R | R | R | S | R | Y | R |

| **GNN** | **Experiment type** | **Finger 1** | | | **Finger 2** | | | **Finger 3** | | |
| --- | --- | --- | --- | --- | --- | --- | --- | --- | --- | --- |
| **GTC** |  | -1 | 3 | 6 | -1 | 3 | 6 | -1 | 3 | 6 |
|  | 1.Exp |  |  |  | D | A | R |  |  |  |
|  | 2. Predictions from throughout | | | | | | | | | |
|  | a. Approach 1 |  |  |  | Q | V | T | D | H | R |
|  | b. Approach 3 |  |  |  | R | H | S | R | Y | R |

| **GNN** | **Experiment type** | **Finger 1** | | | **Finger 2** | | | **Finger 3** | | |
| --- | --- | --- | --- | --- | --- | --- | --- | --- | --- | --- |
| **GCT** |  | -1 | 3 | 6 | -1 | 3 | 6 | -1 | 3 | 6 |
|  | 1.Exp | R | E | R | T | E | R | Q | D | R |
|  |  |  |  |  | Q | D | R | T | E | R |
|  | 2. Predictions from throughout | | | | | | | | | |
|  | a. Approach 1 | T | H | R | Q | S | V | R | S | R |
|  | b. Approach 3 | R | Y | R | R | R | S | R | Y | R |
