# Supplementary material for: Exploiting the recognition code for elucidating the mechanism of zinc finger protein-DNA interactions
Source: BMC Genomics. 2016 Dec 22;17(Suppl 13):1037. doi: 10.1186/s12864-016-3324-8 (PMC5260074; doi:10.1186/s12864-016-3324-8)
Supplement: Additional file 3: Table S3. — Checking top predictions (top 50) to establish relationship between Approach 2 (consensus amino acids and synergistic binding mode) and Approach 1 (consensus amino acid and modular binding mode) prediction for all 16 GNN targets. Our Approach 2 predictions for Finger 3 coincide with the Approach 1 predictions. (DOCX 23 kb) [file 12864_2016_3324_MOESM3_ESM.docx]

### Additional file 3 – Checking top predictions (top 50) to establish relationship between Approach 2 (consensus amino acids and synergistic binding mode) and Approach 1 (consensus amino acid and modular binding mode) prediction for all 16 GNN targets

Our Approach 2 predictions for Finger 3 coincide with the Approach 1 predictions

| **GCG** | **Finger1** | **Finger2** | **Finger3** |
| --- | --- | --- | --- |
| **Approach 1** | QHR (2.4) | RDR (5.5) | RSQ (2.6) |
| **Approach 2** | RNK | RNK | RNK |
| **Approach 1 rank for Approach 2 prediction** | 82 | 136 | 34 |

| **GCC** | **Finger1** | **Finger2** | **Finger3** |
| --- | --- | --- | --- |
| **Approach 1** | ATR(2.78) | RHR (3.06) | NSK (2.77) |
| **Approach 2** | NST | NST | NST |
| **Approach 1 rank for Approach 2 prediction** | 32 |  | 1 |

| **GGG** | **Finger1** | **Finger2** | **Finger3** |
| --- | --- | --- | --- |
| **Approach 1** | RHE (1.99) | THR (2.15) | NHR (4.5) |
| **Approach 2** | RTR | RTR | RTR |
| **Approach 1 rank for Approach 2 prediction** | - | 24 | 14 |

| **GCT** | **Finger1** | **Finger2** | **Finger3** |
| --- | --- | --- | --- |
| **Approach 1** | THK(3.05) | QSV (4.70) | RSR (3.08) |
| **Approach 2** | AAK | AAK | AAK |
| **Approach 1 rank for Approach 2 prediction** | 32 | 49 | 12 |

| **GGC** | **Finger1** | **Finger2** | **Finger3** |
| --- | --- | --- | --- |
| **Approach 1** | RVK(1.55) | DTR (2.94) | HDR (3.4) |
| **Approach 2** | THQ | THQ | THQ |
| **Approach 1 rank for Approach 2 prediction** |  | 26 | 51 |

| **GCA** | **Finger1** | **Finger2** | **Finger3** |
| --- | --- | --- | --- |
| **Approach 1** | RHE (2.9) | DHR (2.07) | HHR (2.5) |
| **Approach 2** | ATK | ATK | ATK |
| **Approach 1 rank for Approach 2 prediction** | 137 | 27 | - |

| **GTG** | **Finger1** | **Finger2** | **Finger3** |
| --- | --- | --- | --- |
| **Approach 1** | ASR | QNQ | TDR |
| **Approach 2** | RAQ | RAQ | RAQ |
| **Approach 1 rank for Approach 2 prediction** | 166 | 334 | 406 |

| **GGA** | **Finger1** | **Finger2** | **Finger3** |
| --- | --- | --- | --- |
| **Approach 1** | QHR | RVR | QHR |
| **Approach 2** | NNR | NNR | NNR |
| **Approach 1 rank for Approach 2 prediction** | 88 | 210 | - |

| **GGT** | **Finger1** | **Finger2** | **Finger3** |
| --- | --- | --- | --- |
| **Approach 1** | AST | NDR | TDR |
| **Approach 2** | NVQ | NVQ | NVQ |
| **Approach 1 rank for Approach 2 prediction** | 158 | 321 | - |

| **GAA** | **Finger1** | **Finger2** | **Finger3** |
| --- | --- | --- | --- |
| **Approach 1** | QHR | RHR | DDT |
| **Approach 2** | DNT | DNT | DNT |
| **Approach 1 rank for Approach 2 prediction** | 93 | 131 | 202 |

| **GTT** | **Finger1** | **Finger2** | **Finger3** |
| --- | --- | --- | --- |
| **Approach 1** | AAR | DTT | RHR |
| **Approach 2** | AHT | AHT | AHT |
| **Approach 1 rank for Approach 2 prediction** | 72 | 167 | - |

| **GAT** | **Finger1** | **Finger2** | **Finger3** |
| --- | --- | --- | --- |
| **Approach 1** | RDA | QSR | RHQ |
| **Approach 2** | HAT | HAT | HAT |
| **Approach 1 rank for Approach 2 prediction** | 356 | - | 365 |

| **GTA** | **Finger1** | **Finger2** | **Finger3** |
| --- | --- | --- | --- |
| **Approach 1** | RDA | QSR | HHR |
| **Approach 2** | QTK | QTK | QTK |
| **Approach 1 rank for Approach 2 prediction** | 12 | 229 | - |

| **GAC** | **Finger1** | **Finger2** | **Finger3** |
| --- | --- | --- | --- |
| **Approach 1** | NHR | TSN | TSR |
| **Approach 2** | RSQ | RSQ | RSQ |
| **Approach 1 rank for Approach 2 prediction** | 230 | 447 | - |

| **GTC** | **Finger1** | **Finger2** | **Finger3** |
| --- | --- | --- | --- |
| **Approach 1** | THV | QVT | DHR |
| **Approach 2** | HSR | HSR | HSR |
| **Approach 1 rank for Approach 2 prediction** | 20 | 48 | 131 |

| **GAG** | **Finger1** | **Finger2** | **Finger3** |
| --- | --- | --- | --- |
| **Approach 1** | AAK | NVK | ADK |
| **Approach 2** | HTN | HTN | HTN |
| **Approach 1 rank for Approach 2 prediction** | 13 | 45 | 10 |
